# Genetic Modification of a *Hox* Locus Drives Mimetic Color Pattern Variation in a Highly Polymorphic Bumble Bee

**DOI:** 10.1093/molbev/msad261

**Published:** 2023-12-01

**Authors:** Wanhu Yang, Jixiang Cui, Yuxin Chen, Chao Wang, Yuanzhi Yin, Wei Zhang, Shanlin Liu, Cheng Sun, Hu Li, Yuange Duan, Fan Song, Wanzhi Cai, Heather M Hines, Li Tian

**Affiliations:** Department of Entomology and MOA Key Lab of Pest Monitoring and Green Management, College of Plant Protection, China Agricultural University, Beijing 100193, China; Department of Entomology and MOA Key Lab of Pest Monitoring and Green Management, College of Plant Protection, China Agricultural University, Beijing 100193, China; Department of Entomology and MOA Key Lab of Pest Monitoring and Green Management, College of Plant Protection, China Agricultural University, Beijing 100193, China; Department of Entomology and MOA Key Lab of Pest Monitoring and Green Management, College of Plant Protection, China Agricultural University, Beijing 100193, China; Department of Entomology and MOA Key Lab of Pest Monitoring and Green Management, College of Plant Protection, China Agricultural University, Beijing 100193, China; State Key Laboratory of Protein and Plant Gene Research, School of Life Sciences, Peking University, Beijing 100871, China; Department of Entomology and MOA Key Lab of Pest Monitoring and Green Management, College of Plant Protection, China Agricultural University, Beijing 100193, China; College of Life Sciences, Capital Normal University, Beijing 100048, China; Department of Entomology and MOA Key Lab of Pest Monitoring and Green Management, College of Plant Protection, China Agricultural University, Beijing 100193, China; Department of Entomology and MOA Key Lab of Pest Monitoring and Green Management, College of Plant Protection, China Agricultural University, Beijing 100193, China; Department of Entomology and MOA Key Lab of Pest Monitoring and Green Management, College of Plant Protection, China Agricultural University, Beijing 100193, China; Department of Entomology and MOA Key Lab of Pest Monitoring and Green Management, College of Plant Protection, China Agricultural University, Beijing 100193, China; Department of Biology, The Pennsylvania State University, University Park, PA 16802, USA; Department of Entomology and MOA Key Lab of Pest Monitoring and Green Management, College of Plant Protection, China Agricultural University, Beijing 100193, China

**Keywords:** bumble bees, Müllerian mimicry, noncoding RNA, *Hox*, genomic hotspot

## Abstract

Müllerian mimicry provides natural replicates ideal for exploring mechanisms underlying adaptive phenotypic divergence and convergence, yet the genetic mechanisms underlying mimetic variation remain largely unknown. The current study investigates the genetic basis of mimetic color pattern variation in a highly polymorphic bumble bee, *Bombus breviceps* (Hymenoptera, Apidae). In South Asia, this species and multiple comimetic species converge onto local Müllerian mimicry patterns by shifting the abdominal setal color from orange to black. Genetic crossing between the orange and black phenotypes suggested the color dimorphism being controlled by a single Mendelian locus, with the orange allele being dominant over black. Genome-wide association suggests that a locus at the intergenic region between 2 abdominal fate-determining *Hox* genes, *abd-A* and *Abd-B*, is associated with the color change. This locus is therefore in the same intergenic region but not the same exact locus as found to drive red black midabdominal variation in a distantly related bumble bee species, *Bombus melanopygus*. Gene expression analysis and RNA interferences suggest that differential expression of an intergenic long noncoding RNA between *abd-A* and *Abd-B* at the onset setal color differentiation may drive the orange black color variation by causing a homeotic shift late in development. Analysis of this same color locus in comimetic species reveals no sequence association with the same color shift, suggesting that mimetic convergence is achieved through distinct genetic routes. Our study establishes *Hox* regions as genomic hotspots for color pattern evolution in bumble bees and demonstrates how pleiotropic developmental loci can drive adaptive radiations in nature.

## Introduction

Understanding what and how changes in the genome can lead to novel phenotype is important for understanding the origin of biodiversity (Reed et al. 2011; Wang et al. 2022). Convergent phenotypes are of particular value in addressing these questions as they provide the natural replicates of the evolutionary process necessary to uncover predictable trends of evolution, such as whether same genes or genomic regions are targeted during the evolution of similar traits (Onuma et al. 2002; Mundy 2005; Hines and Rahman 2019; Van Belleghem et al. 2023), whether functional genomic changes primarily occur in the coding or regulatory regions of target genes (Martin and Reed 2010; Reed et al. 2011; Westerman et al. 2018; Lewis et al. 2019), and whether certain types of genes are evolutionary hotspots (Baxter et al. 2008; Martin and Orgogozo 2013). In particular, Müllerian mimicry, where 2 or more distasteful sympatric species exhibit similar aposematic signals to decrease the risk of predation (Müller 1879), is an exceptional case of phenotypic divergence and convergence. Decades of research on the wing pattern mimicry of the Neotropical *Heliconius* butterflies have provided numerous insights into the genomic mechanisms driving phenotypic evolution, demonstrating that adaptive evolution is commonly driven by a few major effect loci, and divergent lineages can repeatedly use the same genes to generate similar phenotypes (Reed et al. 2011; Nadeau et al. 2016; Mazo-Vargas et al. 2017; Westerman et al. 2018).

Bumble bees present another striking example of color pattern diversification driven by Müllerian mimicry (Williams 2007; Hines and Williams 2012; Rapti et al. 2014). Female bees display bright, contrasting color bands imparted by dense body setae (also known as setal pile, Rapti et al. 2014) to signal their toxic sting (Brower et al. 1960). Unrelated sympatric species may share a common aposematic color pattern to enhance their warning signal, forming multispecies mimicry complexes (Williams 2007). In addition to interspecific mimicry, many species display intraspecific geographical polymorphism, with different geographic populations of a single species displaying distinct color patterns as a result of converging on regional mimetic patterns (Plowright and Owen 1980; Hines and Williams 2012; Huang et al. 2015). The rampant regional convergence and cross-regional divergence has helped generate over 400 color patterns across the ∼270 bumble bee species (Williams 2007). Although multiple ecological factors are proposed for driving color pattern variation in these bees, Müllerian mimicry is believed to contribute to most of this diversity (Williams 2007; Tian et al. 2019).

The color patterns of bumble bees occur in a highly modular, segmental fashion (Williams 2007; Rapti et al. 2014), with color tending to shift by segmental sclerites of the head, thorax (mesosoma), and abdomen (metasoma). Polymorphic species obtain multiple color patterns by shifting colors of particular segments without changing other body regions (Plowright and Owen 1980; Hines and Williams 2012; Tian et al. 2019). Rapti et al. (2014) quantitatively analyzed color patterns across bumble bees to discover 12 discrete “ground plan” color pattern elements. The boundaries of these elements correspond well to the boundaries of expression domains of segmental fate-determining *Hox* genes in insects, suggesting that these upstream selector genes may play a key role in segmental color patterning (Rapti et al. 2014).

The genetic basis of color pattern polymorphism has been studied in the North America species, *Bombus melanopygus* Cresson (Owen and Plowright 1980; Plowright and Owen 1980; Tian et al. 2019). The species switches its midabdominal (T2-3; supplementary fig. S1, Supplementary Material online) tergal pile color discretely from red to black between the Pacific Coastal and the Rocky Mountain regions to participate in distinct regional mimetic complexes. Classical genetic crossing revealed the 2 color morphs to comprise a single species and to be controlled by a single Mendelian locus, with red being dominant to black (Owen and Plowright 1980). This shift involves a modification of melanin types from red phaeomelanin to black eumelanin, the same color switch involved in red and black hair coloration in vertebrates (Hines et al. 2017). Although a pigmentation gene would then be a good candidate (Jeong et al. 2006; Kronforst et al. 2012), using genome-wide association analysis (GWAS) and gene expression data, Tian et al. (2019) revealed the color dimorphism in *B. melanopygus* to be controlled higher up in the developmental network—by *cis*-regulatory mutations that alter the timing (heterochrony) and location (heterotopy) of expression of a *Hox* gene, *Abdominal-B* (*Abd-B*). *Hox* genes are primed for controlling segmental color variation because they are the major selector genes for segmental phenotypes (Akam 2002; Hughes and Kaufman 2002; Maeda and Karch 2009). However, shifting *Hox* gene expression can have major homeotic and pleiotropic effects in controlling fate of many segmental phenotypes (Bender et al. 1983; Brena et al. 2005). Tian et al. (2019) found that shifting gene expression late in development likely allowed these genes to perform micromanaging roles in driving color, which develops near the point of adult eclosion (Tian and Hines 2018; Rahman et al. 2021), without modifying other aspects of morphology. Further research is needed to determine whether *Hox* genes may play a more universal role in bumble bee coloration and whether *Abd-B* performs this role ancestrally.

**Fig. 1.**
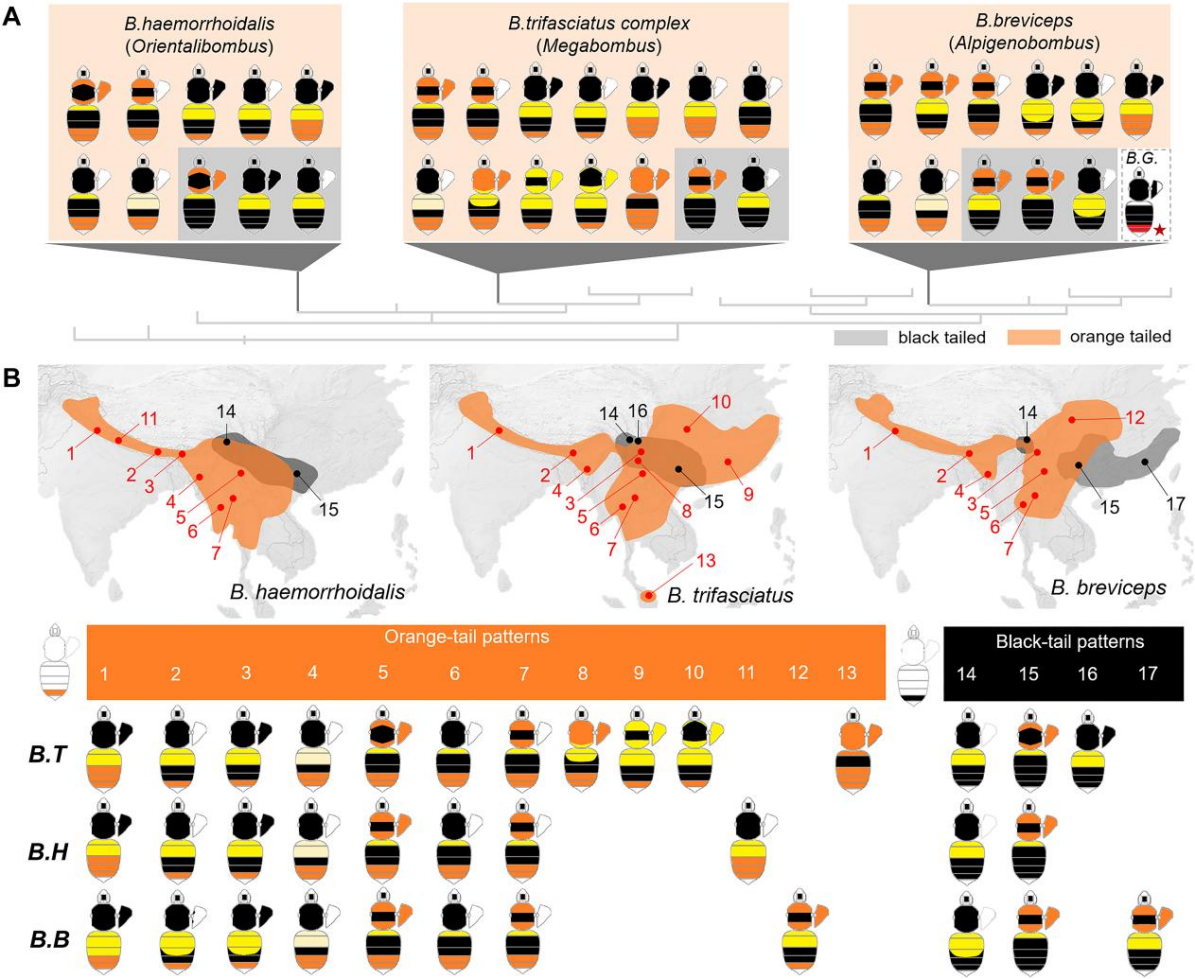
Mimetic color pattern diversity of the South Asia bumble bee mimicry group. A) Phylogenic position and color pattern diversity of the comimetic and distantly related *B. breviceps* (*B. B*), *B. haemorrhoidalis* (*B. H*), and *B. trifasciatus* (*B. T*) lineages, belonging to subgenera *Alpigenobombus*, *Orientalibombus*, and *Megabombus*, respectively. Note that this mimetic group displays parallel color variation on different body segments including thoracic, pleuron, anterior metasomal tergal (T1-3), and finally, 5th metasomal tergal (T5, also known as “tail” in bumble bees) regions, which displays the orange/black dimorphism studied here. All mimetic color patterns of these lineages are divided into 2 groups based on color phenotype (orange or black) on metasomal T5, highlighted by orange and gray shading, respectively. The color pattern diagram with a red star represents *B. grahami* (*B. G.*), the sister species of *B. breviceps*. B) Major mimetic color patterns of the 3 lineages and their simplified geographic distribution. Dots and connecting numbers on the map indicate different locations where mimicry among the 3 lineages cooccur. Graphs below the maps illustrate major mimetic color patterns in those respective locations by comimetic species.

To further understand the genetic basis of bumble bee mimetic variation, we explore color pattern variation in bumble bee mimicry complexes of South Asia. Multiple regionally distinct mimicry complexes occur here and are displayed primarily by 3 subtropical lineages: *Bombus* (*Orientalibombus*) *haemorrhoidalis* Smith, *Bombus* (*Megabombus*) *trifasciatus* species complex, and *Bombus* (*Alpigenobombus*) *breviceps* Smith (Fig. 1; Hines and Williams 2012). These phylogenetically distant lineages (Cameron et al. 2007) have a combined range from the western Himalayas to the southeast coast of China and south to Malaysia. Across the sympatric regions, they have converged on over 10 different mimetic color patterns (Hines and Williams 2012). The color pattern diversity of these bees comprises discrete variations on different body regions (Fig. 1), with pile color variation occurring on the thoracic dorsum, pleuron, and metasomal tergites, changing among orange, black, white, and yellow (Fig. 1; supplementary fig. S1, Supplementary Material online). The richness of segmental variation within species and striking convergence of segmental color patterns between species makes the Southeast Asian groups ideal systems to explore genetic mechanisms driving mimetic diversification in bumble bees.

We begin this exploration by deciphering the genetic basis of color pattern variation in *B. breviceps*. This species is one of the most abundant species in southern China, where it has great potential for domestication for greenhouse pollination and is the subject of extensive research (Zhao et al. 2017; Liang et al. 2020; Sun et al. 2020; Ren et al. 2023). To date, the species has been successfully reared under laboratory conditions (Liang et al. 2020) and its chromosome-level genome assembly has been completed (Sun et al. 2020), making it well-suited for developmental genetic studies. In particular, we decipher the genetics basis of the dimorphic orange black shift in the posterior “tail” region (abdominal tergite T5; Fig. 1 and supplementary fig. S1, Supplementary Material online) just anterior to the sting, sampling the orange-tailed phenotypes known to occur in the southwestern China highlands and the black-tailed populations in the lower mountainous regions of southeast China (Fig. 1; Hines and Williams 2012).

Toward these goals, we perform GWAS and population-wide genotyping to successfully reveal the genomic locus controlling this dimorphism. Using gene expression analysis across critical setal pigmentation stages as well as functional validation by RNA interference, we further refine the molecular mechanisms by which the locus directs color variation. In addition, we examine the implicated locus in comimetic species *Bombus* (*trifasciatus*) *montivagus* and *B. haemorrhoidalis* to determine whether convergent phenotypes are regulated by the same mutations. Our results expand our knowledge on how a highly pleiotropic developmental locus can be targeted to drive phenotypic variation, expanding our genetic understanding of the exceptional color diversity in these bees.

## Results

### The Orange Black Genomic Locus Is Located at the Intergenic Region of 2 *Hox* Genes of the Bithorax Complex

**Fig. 2.**
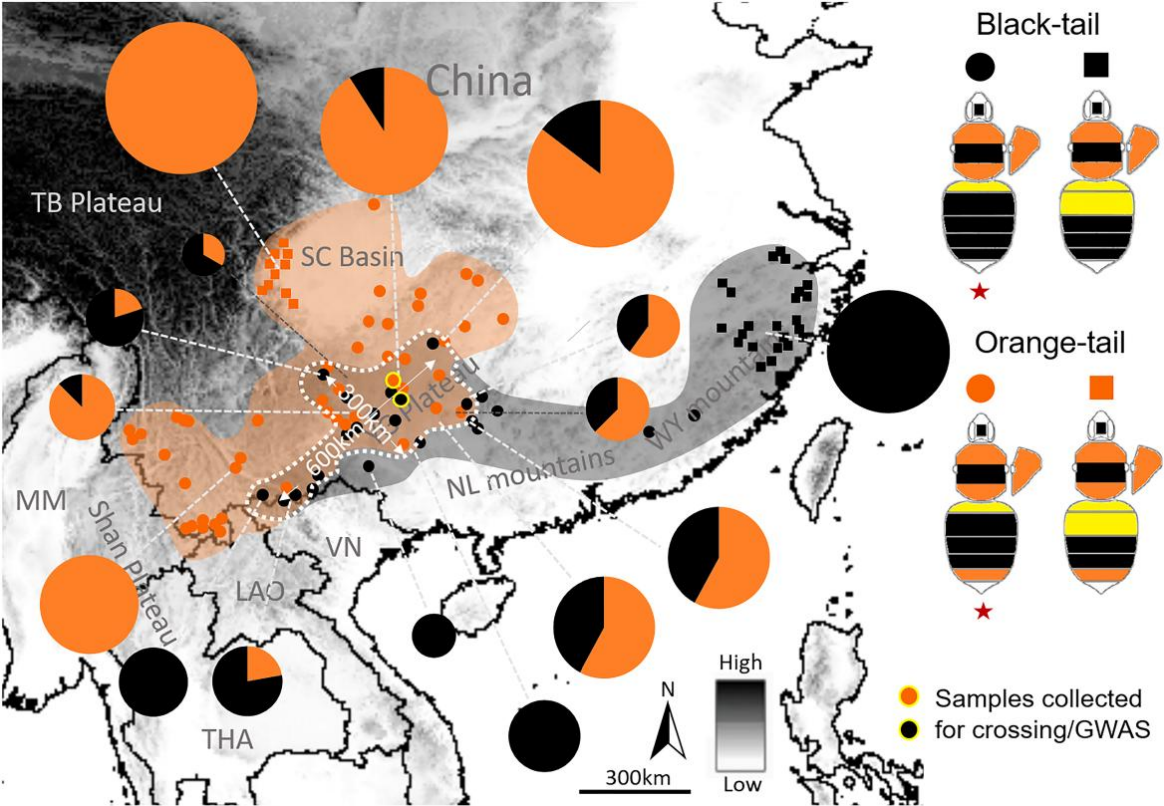
The Southern China orange/black color pattern transition zone of *B. breviceps*. Dots and squares on the map indicate localities from where bees were collected and phenotyped. The color pattern transition zone is highlighted by dashed white circles with distances labeled. Note that this transition zone is ∼300 km in width from northwest to southeast and 600 km in length from northeast to southwest, involving southern Yunnan, all of Guizhou, and southern Hunan. This transition zone occurs across altitudinal gradients, with the orange/black ratio gradually decreasing from northwest to southeast, largely concordant with the topographic transition from the Yun-Gui highlands to the lower elevation mountainous area of southeastern China. Orange and black color indicates orange- and black-tailed patterns, respectively. Pie charts indicate frequency of orange- and black-tailed patterns at each location, represented by percentage of orange-/black-tailed bees in the total collection from that location (supplementary data, Supplementary Material online). The size and portion of these charts reflect the number of samples per locality. Dots with a yellow circle indicate locations where samples were collected for GWAS and genetic crossing. The orange black transitions occur across an altitudinal gradient. Across the collection regions, each of the orange- and black-tailed patterns display variations on yellow banding numbers on anterior abdominal terga (T1-2), based on which each of them can be further partitioned into 1 yellow-banded and 2 yellow-banded color pattern groups, indicated by dots and squares, respectively. However, only the 1 yellow-banded pattern (indicated by red stars) of the orange/black phenotypes occur in the transition zone and was sampled for GWAS. Dots with yellow circles indicate sites where queens of the offspring males used for GWAS were collected. MM, Myanmar; THA, Thailand; LAO, Laos; VN, Vietnam; TB, Tibet Plateau; SC, Sichuan Basin; YG, Yunnan-Guizhou Plateau; NL, Nanling Mountains; WY, Wuyi Mountains.

**Fig. 3.**
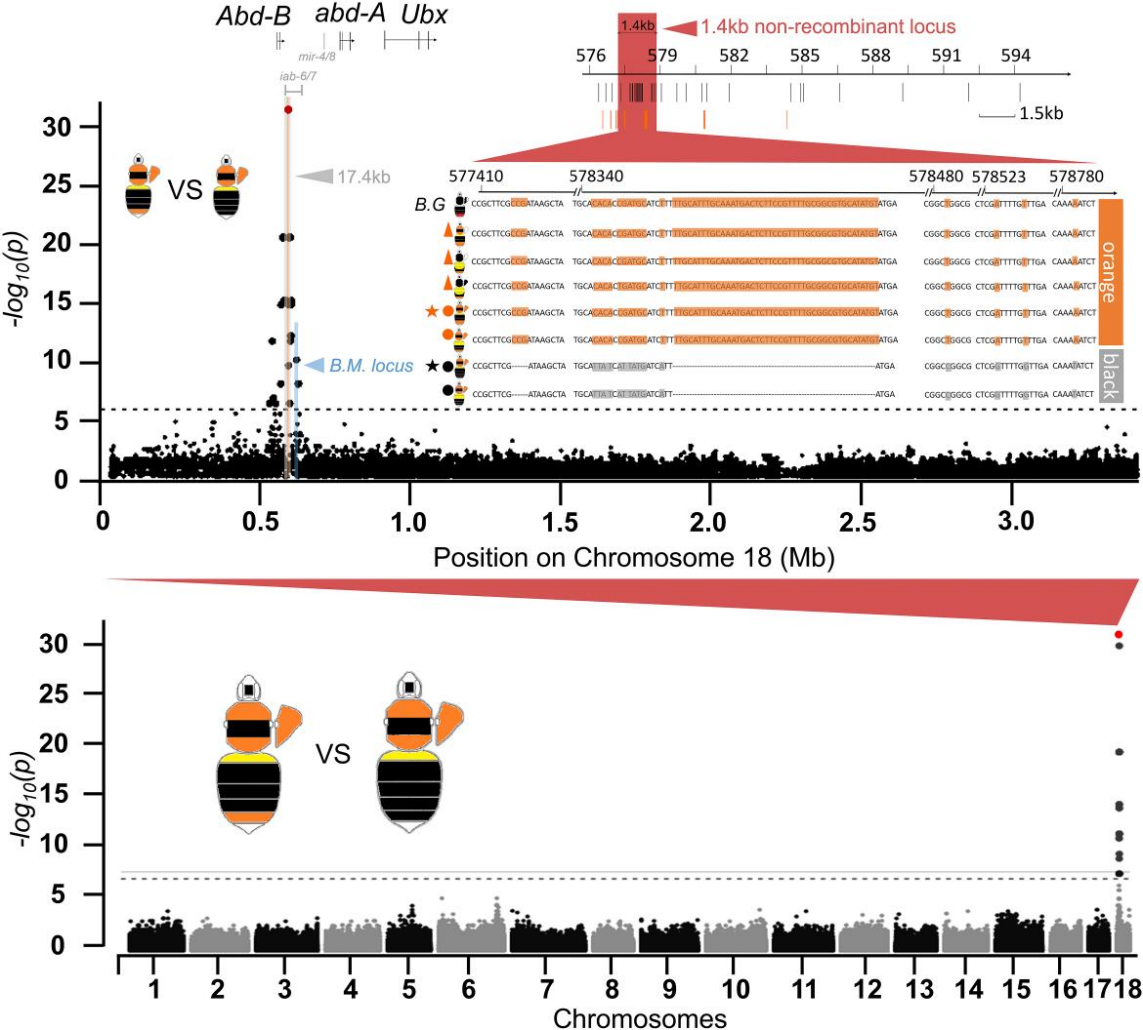
The color locus revealed by GWAS. The Manhattan plot on the bottom is the whole-genome association showing that sites of fixed association (indicated in red) are confined to a single locus on chromosome 18. The plot above shows association within this chromosome, showing that fixation is confined to a ∼17.4 kb block 3′ of *Abd-B* (highlighted gray). The region highlighted in red indicates the position of the 1.4 kb nonrecombinant region that remains fixed with additional genotyping. The region highlighted in blue indicates the color locus controlling the red/black midabdominal color dimorphism in *B. melanopygus* (Tian et al. 2019). SNPs (black bars) and indels (orange bars) and their position are indicated below the 1.4 kb locus. Note that the black pattern contains 3 and 46 bp deletions at the nonrecombinant locus. The sequences show genotypes of different orange and black color patterns at the 1.4 kb locus, as well as that of *B. grahami* (B.G.) with only partial fragments containing the fixed SNPs and indels shown. Stars indicate patterns used for GWAS. Dots indicate color patterns used for narrowing the locus. Triangles indicate additional geographic color pattern genotypes for validating the association of this 1.4 kb locus with color morphs. Note that the locus remains fixed across all orange- and black-tailed patterns regardless of their geographic locations and variations on other segmental phenotypes (e.g. thorax and pleuron).

Color pattern distribution analysis on georeferenced specimens (supplementary methods, Supplementary Material online) located a phenotypic transition zone of the orange/black tail in Yunnan-Guizhou plateau of southern China (Fig. 2). Our examination of color pattern inheritance based on phenotypic segregation among progeny of wild-caught queens collected from this transition zone, as well as those from controlled genetic crosses (supplementary methods, Supplementary Material online), suggests that the orange/black dimorphism in *B. breviceps* is controlled by a single, biallelic Mendelian gene, with the orange (R) allele dominant to the black (r) (supplementary fig. S2 and tables S1 and S2, Supplementary Material online). Based on these results, orange- and black-tailed male progeny from colonies established by queens collected from the phenotypic transition zone (Fig. 2) was used for GWAS. Transition zones are ideal for genotype–phenotype association analysis to locate the locus driving trait variation because different phenotypes in the transition zone have experienced natural hybridization and gene flow (Mallet 1993; Mavárez et al. 2006), neutralizing genetic population structure that would potentially cause strong background noise during association analysis (Jiggins et al. 1996; Reed et al. 2011). In line with the inheritance data, GWAS revealed a single fixed ∼17 kb peak of association on the 18th chromosome (Fig. 3), in an intergenic domain (also known as the *iab* regions; Fig. 3) between *abd-A* and *Abd-B*, 2 *Hox* genes of the bithorax complex (BX-C) known to control developmental fate of abdominal segments (Lewis 1978). Fine-scale genotyping of 41 black and 51 orange individuals across this block further narrowed the associated region to a 1.4 kb, nonrecombinant interval, containing 2 insertions and deletions (indels) (a short 3 bp one and a longer 46 bp one) and 14 single nucleotide polymorphisms (SNPs), closest to and ∼25 kb downstream from the *Abd-B* (Fig. 3). Aligning the *abd-A/Abd-B* region between *B. breviceps* and *Drosophila* (Tian et al. 2019) suggested that the relative position of the *B. breviceps* color locus also corresponds to the *Drosophila iab-6/7* region, which is very close to the *B. melanopygus* locus (∼25 kb upstream from the locus; Fig. 3; supplementary fig. S3, Supplementary Material online).

**Fig. 4.**
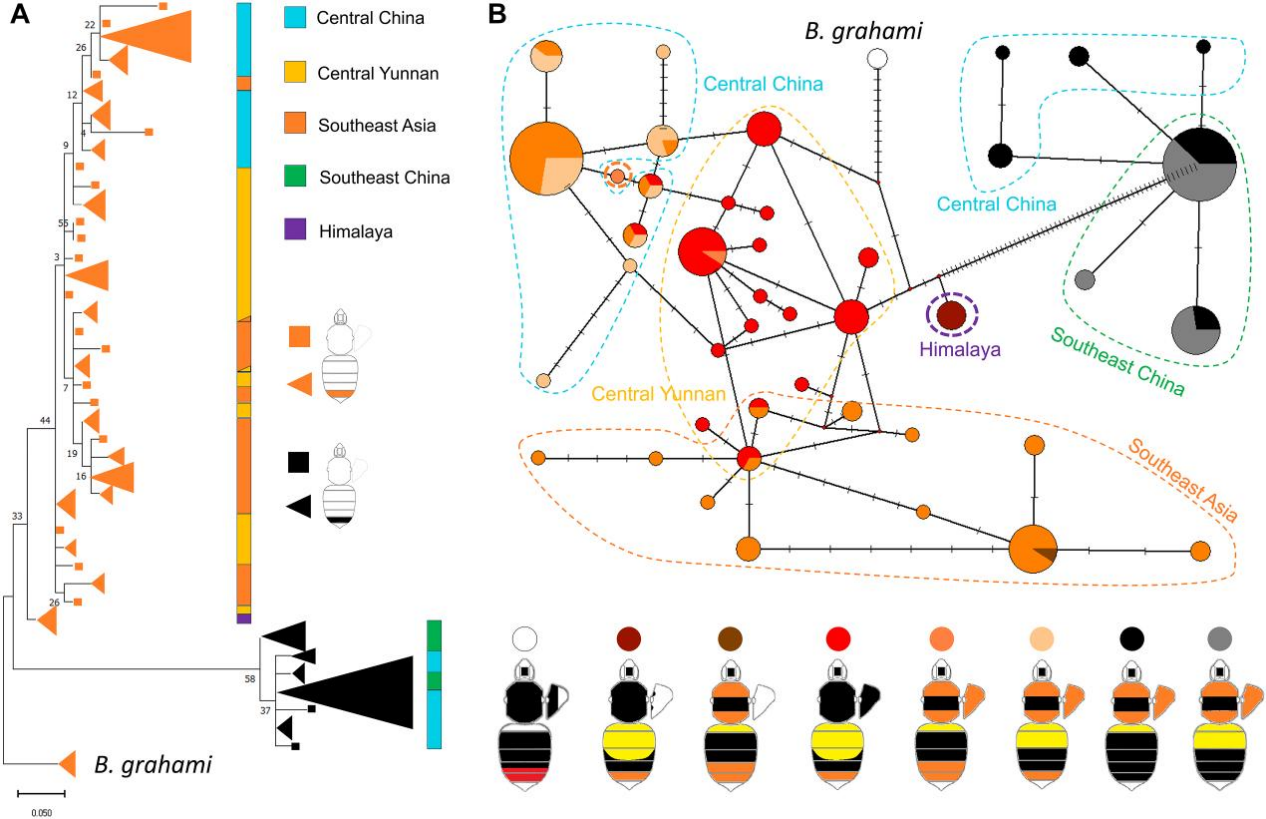
Phylogeographic relationships of different color pattern lineages of *B. breviceps.* A) Phylogenetic clustering of color pattern at the 1.4 kb color locus. Trees were constructed using ML methods with terminal nodes colored by the 2 tail color phenotypes. Triangular clades represent shared haplotypes, with size reflecting the frequency of the haplotype. Colored bars indicate broad geographic regions. B) Haplotype networks of the color locus. Each node represents a haplotype, lines between nodes represent a single base change, and the size of the node represents haplotype frequency. Nodes are colored by the color patterns containing that haplotype. Geographic regions are indicated using dashed polygons.

Population-wide genotyping revealed that the 1.4 kb locus is associated with the orange/black tail phenotype across all surveyed color patterns and geographic populations of *B. breviceps* (Fig. 3). In addition, both phylogenetic reconstruction (Fig. 4A) and network analysis (Fig. 4B) for haplotypes of the 1.4 kb locus showed strong structure by phenotype and separated the orange- and black-tailed patterns into separate monophyletic clades. Within each clade, there is also substantial haplotype clustering by geography. These results further support the tight association of the color locus with the phenotypes. Interestingly, the orthologous locus of the monomorphic sister species of *B. breviceps*, *Bombus grahami*, which has orange in metasomal T5, carries all fixed indels and SNPs of the orange genotypes on the homologous 1.4 kb locus (Fig. 3), suggesting that the orange genotype of the *B. breviceps* is inherited from the common ancestor of *B. breviceps* and *B. grahami* and the black genotype is derived within the *B. breviceps* populations (but see supplementary results, Supplementary Material online for more details).

### The Orange Black Color Shift Is Not Associated with Significant Differential Expression of *abd-A* and *Abd-B*

**Fig. 5.**
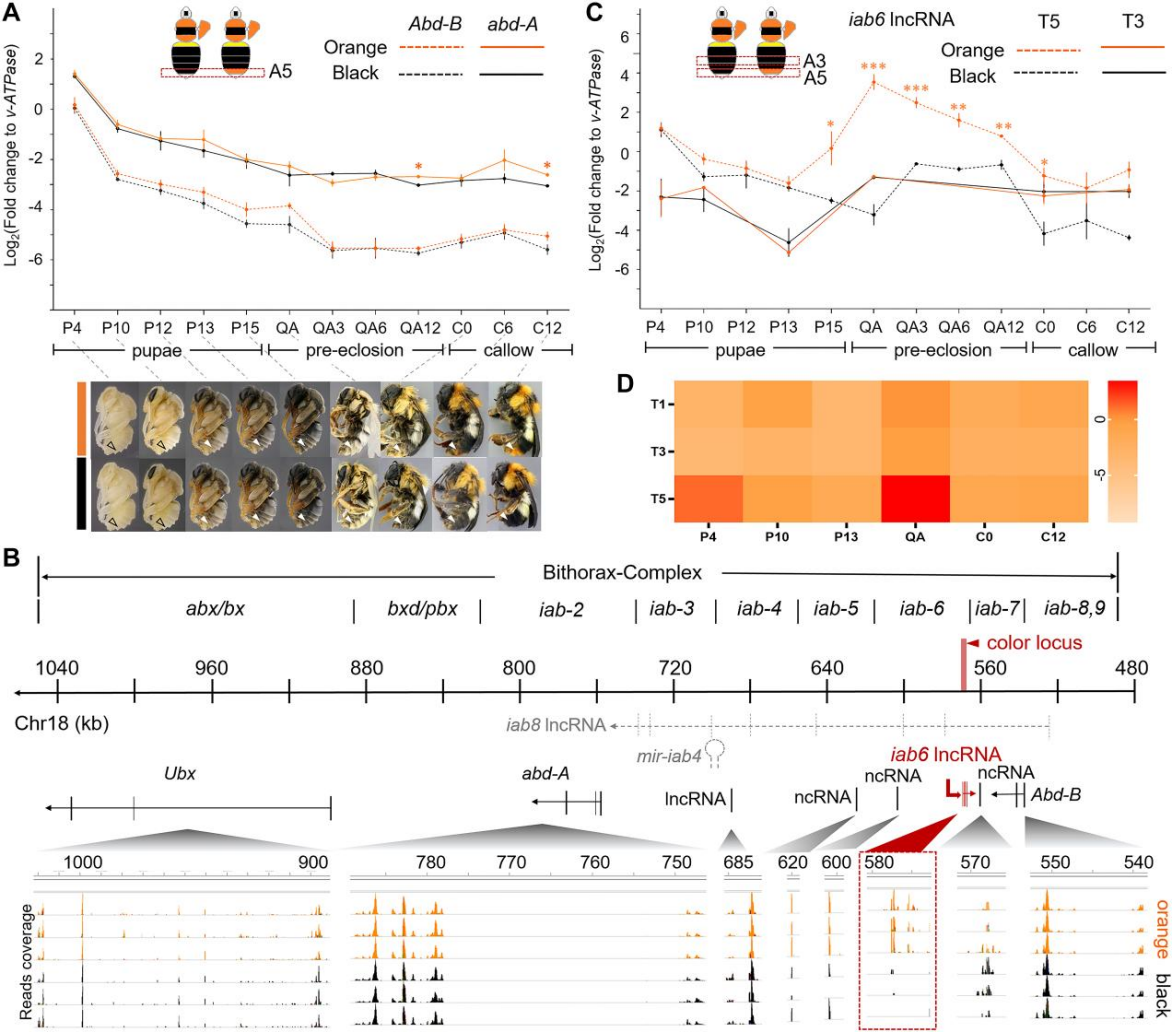
Gene expression analysis for the bithorax complex (BX-C) during bumble bee development. A) Temporal expression pattern of the *abd-A* and *Abd-B* genes at the dimorphic metasomal tergal segment 5 (T5) across pupal and early adult development. Images for pupal (P4-P15) and adult (QA-C12) stages used for qPCR analysis are provided below the gene expression plot. These images also demonstrate the process of setal pigmentation. The white arrows indicate polymorphic metasomal T5. Note that the orange and black setae are distinguishable starting from QA stage. At the QA stage, black setae exhibit a grayer tinge, while the orange setae become more golden orange. The 2 phenotypes become more distinguishable at the time of eclosion (0 h callow). Color continues to intensify in the first 24 h posteclosion. Both orange and black setae attain most of their color within the first 6 h of posteclosion and complete pigmentation by 12–24 h, largely in line with what has been found in other bumble bees (Tian and Hines 2018). B) The gene transcription profile of orange- and black-tailed bees across the BX-C complex at the QA period. The numbered line in the middle represents position of the chromosomal 18 corresponding to the BX-C complex. The red bar indicates the color locus. Positions of the infra-abdominal (*iab*) domain are labeled above the line. Gray dashed lines immediately below the chromosomal line indicate position of the *iab8* lncRNA and *mir-iab4* microRNA discovered by previous studies. Black solid lines indicate position of the protein coding sequence of BX-C *Hox* genes, as well as ncRNAs recognized by current studies. The *iab6* lncRNA were shown in red solid line. The bottom represents transcriptome sequencing reads that mapped to the BX-C regions, with the height of the peak indicating read coverage. Each line represents the transcription profile of an individual bee, with orange- and black-colored graphs representing orange-tailed and black-tailed bees, respectively. Read coverage of the *iab6* ncRNA is highlighted by a dashed square. Note that this lncRNA only has read coverage in the orange-tailed bees but little to no read coverage in the black-tailed samples, suggesting its tight association with the orange pigmentation. C) Gene expression of *iab6* lncRNA in the dimorphic T5 and monomorphic metasomal T3. D) Heatmap indicating *iab6* lncRNA expression level across abdominal tergites and pupal stages of *B. breviceps*. Note that this lncRNA is highly stage- and tissue-specific, with peak expression at T5 at the QA stage. Error bars in (A) and (C) indicate standard error (SE) of the mean. Stars above the error bars indicate levels of statistical significance *P < 0.05, **P < 0.01, ***P < 0.001 by 2-sample *t*-test.

Previous studies have demonstrated that the *iab* region is subdivided into several noncoding domains (*iab*2-9) (Lewis 1978; Bae et al. 2002) that exercise regulatory function to direct expression of *abd-A* and *Abd-B* (Sanchez-Herrero 1991; Akbari et al. 2006; Long et al. 2017). The *B. melanopygus* color locus, which falls in the *iab6/7* region, controls color variation via regulating *Abd-B* expression (Tian et al. 2019; Rahman et al. 2021). Given the much closer vicinity of the *B. breviceps* locus to *Abd-B*, it is most likely also directing setal pigmentation via regulating expression of this gene. To test this, we performed quantitative real-time PCR (qRT-PCR) to investigate expression of *abd-A* and *Abd-B* in the epidermal tissues of the dimorphic metasomal T5 across different stages of setal development. Examination of the setal pigmentation process for *B. breviceps* suggested that the orange/black color differentiation on metasomal T5 begins at the pre-eclosion, quiescent adult (QA) stage (Fig. 5A), a point at which bees have shed their pupal cuticle but have not yet emerged from its cocoon (Tian and Hines 2018). Both orange and black setae complete pigmentation by 12–24 h after eclosion (12∼24 h callow; Fig. 5A). These observations suggest critical molecular and physiological processes related to color differentiation occur around late pupal and early adult stages.

We sampled the epidermis of T5 from worker bees across pupal–adult stages for qPCR analysis. The results demonstrate that the mRNA level of these *abd-A* and *Abd-B* were highest in early pupal stages (e.g. P4) and gradually decrease during pupal development. In addition, *abd-A* showed higher mRNA expression than *Abd-B* at T5, a pattern consistent with previous findings in insects (Jin et al. 2019). However, neither genes show significant mRNA level differences between phenotypes across most developmental stages (Fig. 5A). A differential expression of *abd-A* between the 2 phenotypes was observed at the 12 h QA stage (QA12), whereby the mRNA level of the black-tailed bees was slightly (∼2×) higher than the orange-tailed bees (*P* = 0.003; 2-sample *t*-test, 2-tailed) (Fig. 5A). However, this upregulation was very mild and occurred after setal color differentiation had begun (e.g. QA stage) (Fig. 5A), thus may not be the initiator of color differentiation. *Abd-B* appears to show a slight elevation in the relevant stages for coloration (P15–QA), but this effect size is small (∼2×) and showed no statistical significance with our current sampling (*P* = 0.16 for P15 and *P* = 0.11 for QA stage, 2-sample *t*-test, 2-tailed). Overall, gene expression analysis suggested that the orange black color shift is not associated with differential gene expression of *abd-A* and may result in differences in *Abd-B*.

### The Orange Black Color Shift Is Associated with Differential Expression of an Intergenic RNA

The *iab* domains are known to direct *abd-A* and *Abd-B* expression in 2 ways. First, some domains may contain *cis-*regulatory sequence that bind with upstream transcription factors to activate or repress *Hox* gene transcription (Sanchez-Herrero 1991; Von Allmen et al. 1996; Akbari et al. 2006). In addition, some regions transcribe into noncoding RNAs (ncRNA) to regulate the transcription and translation of *Hox* genes (e.g. *iab-8* ncRNA and *mi-iab4* microRNA, Bender et al. 1983; Bae et al. 2002; Ronshaugen et al. 2005; Gummalla et al. 2012). Given the lack of mRNA level difference of *Abd-B* and *abd-A*, we asked if this locus corresponds to any regulatory ncRNAs that may direct color differentiation in other ways. We performed transcriptomic analysis on orange and back epidermal tissues of the dimorphic T5 at the QA stage, corresponding to the beginning of color differentiation. We specifically looked for reads mapped to the BX-C region to check if there are any transcripts corresponding to the color locus or nearby genomic regions that are differentially expressed between color phenotypes. Surprisingly, we discovered reads mapped to 4 genomic regions downstream and partially overlapped with our color locus where read coverage is dramatically higher in orange-tailed individuals than the black individuals (Fig. 5B). Reverse transcription (RT)-PCR and Sanger sequencing revealed a single long noncoding RNA (lncRNA) that is at least 383 bp in length (ncRNAs larger than 200 bp is defined as lncRNA) that spans at least 4 exons and overlaps with the 3 bp indel region of the inferred color locus (supplementary fig. S3, Supplementary Material online). The coverage difference between orange and black bees is not due to alignment effects resulting from sequence difference between the 2 phenotypes, because the reference genome was sequenced from a black-tailed individual whereas the expression is higher for the more divergent orange form (Sun et al. 2020). RT-PCR sequences support both phenotypes containing this lncRNA in the same domains, and the only variation in size is that the black form lacks the 3 bp indel sequence (supplementary fig. S3, Supplementary Material online). This fragment falls into the previously reported *iab8* lncRNA in insects (Gummalla et al. 2012; Karch and Maeda 2023), but the transcription direction of this lncRNA is opposite to the *iab8* lncRNA (Fig. 5B), suggesting that it is part of a novel lncRNA. Since its relative position approximately corresponds to the *Drosophila iab-6* region, we refer to this intergenic RNA as *iab6* lncRNA.

To further investigate the mode of expression of the *iab6* lncRNA, we performed qRT-PCR for this transcript for epidermal tissues of metasomal T5 across pupal and callow stages (Fig. 5C). In both orange and black bees, the *iab6* lncRNA is expressed at early pupal stages (P4) and gradually decreases during pupal development and reaches the lowest level in late pupal stages (P13) (Fig. 5C). Between P4 and P13, the orange- and black-tailed bees showed no differential expression of the lncRNA (Fig. 5C). Dramatic differences in its expression, however, began to appear at P15 stage, where the *iab6* lncRNA is significantly upregulated in the orange-tailed bees while continuing to decline in the black-tailed bees (Fig. 5C). In the orange-tailed bees, the expression level of *iab6* lncRNA peaked at early QA stage and started to decline again during subsequent QA (3, 6, and 12 h QA stages) and early callow stages (0, 6, and 12 h callow stages) (Fig. 5C). The peak differentiation of *iab6* lncRNA expression between the orange and black bees was observed at the QA stage, where a mean relative level of *iab6* lncRNA in the orange-tailed bees was dramatically (∼128×) higher (*P* = 0.0005, 2-sample *t*-test, 2-tailed) than the black-tailed bees (Fig. 5C). To exclude the possibility that the difference of the *iab6* lncRNA level between orange- and black-tailed bees represents interpopulation differences rather than being associated with pile colors, we examined its expression at metasomal T3 epidermal terga of orange and black bees, which showed no color differentiation, having a black color in both the orange and black bees. qRT-PCR revealed consistently low-level expression and no differential expression of *iab6* lncRNA between orange and black bees on metasomal T3 tissue across all surveyed stages (Fig. 5C), supporting its role in generating the dimorphic setal color changes. To further validate the functional specificity of this *iab6* lncRNA, we characterized its expression patterns in epidermal tissues of abdominal terga across segments and pupal/adult developmental stages, in the orange-tailed form, using qRT-PCR (Fig. 5D). The results revealed that *iab6* lncRNA is specifically expressed in epidermal tissues of the orange-colored metasomal T5 and has spikes of upregulation at early pupal (P4) (mild upregulation) and QA stages (strong upregulation) (Fig. 5C and D), which correspond to the stages of setal cell differentiation and setal pigmentation, respectively (Hines et al. 2022). This provides further support that the *iab6* lncRNA functions specifically in posterior metasomal segments and its function during pupal stages is primarily associated with setal development and pigmentation. RNA interference (RNAi) targeting the *iab6* lncRNA, which successfully repressed the expression of this lncRNA (∼8 fold knockdown, *P* < 0.05, one-way analysis of variance [ANOVA] with Turkey's honest significant difference [HSD] post hoc test; supplementary fig. S4, Supplementary Material online) at the QA stage, resulted in a shift of setal color on part of the T5 from orange to white or black in 24 h callows (supplementary fig. S4, Supplementary Material online). This further supports the essential role of the *iab6* lncRNA for maintaining the orange pigmentation and for driving the orange black color shift.

### Comimetic Species Use Different Mutations to Achieve Similar Phenotypes

**Fig. 6.**
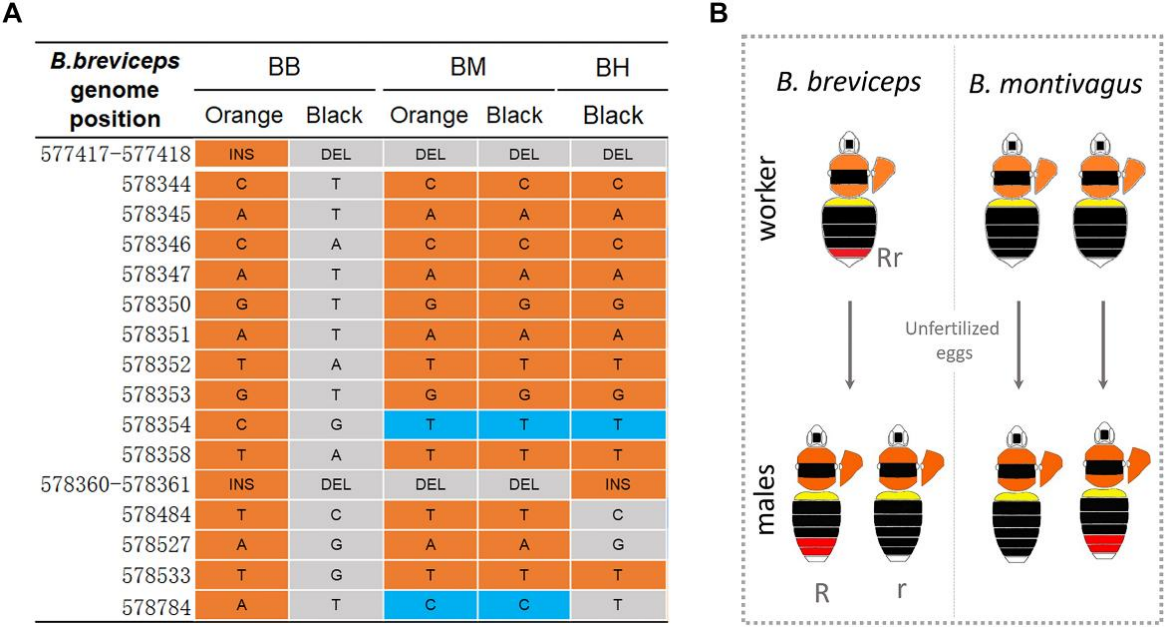
Genetics and inheritance of orange/black dimorphism in *B. breviceps* comimics. A) Allelic variants in the color locus of *B. breviceps* (BB) and the comimetic *B. montivagus* (BM) and *B. haemorrhoidalis* (BH). Orange and gray shading indicates SNPs of the orange- and black-tailed form, respectively. Blue shading indicates alternative SNPs to those in *B. breviceps.* In *B. montivagus*, no fixed SNP or indels are similarly differentiated by color forms. In *B. haemorrhoidalis*, only black-tailed phenotypes were sampled and genotyped, and only a few sites of these black formed bees share the same alleles with the black-tailed *B. breviceps*. B) The left graph shows color patterns of males produced by 2 black-tailed *B. montivagus* workers. One black-tailed *B. montivagus* worker produced orange-tailed males, which is not consistent with the *B. breviceps* inheritance model in which black tails are controlled by recessive alleles and black workers are homozygous for rr and can produce only black r males. This indicates that the orange/black dimorphism in metasomal T5 is governed by different inheritance rules in *B. montivagus*.

To test whether the same dimorphism sported by comimetic species is associated with the same mutations, we compared sequences of the fixed color locus from the orange- and the black-tailed individuals of comimetic species, *B.* (*trifasciatus*) *montivagus* and *B. haemorrhoidalis* (Fig. 6A). In *B. montivagus*, no SNPs or indels that are fixed for *B. breviceps* are similarly differentiated by color forms. In addition, both morphs share the 3 bp and 46 bp deletions exhibited by the black *B. breviceps*. Due to sampling limitations, we only examined the locus for black-tailed *B. haemorrhoidalis*. The black morphs examined carry orange genotypes for 10 out of the 15 SNPs, as well as the 46 bp insertion, but contain the 3 bp black deletion. In summary, these results suggest that the *B. breviceps* color locus is not associated with parallel color shift in comimics.

In addition to genotyping, we also assessed the tail color inheritance of *B. montivagus* using a limited number of wild-caught *B. montivagus* workers (Fig. 6B). Because bumble bee workers can lay unfertilized eggs which would produce male offspring, we caught black-tailed *B. montivagus* workers from the wild and reared them in the lab to observe phenotype segregation on their male offspring. In *B. breviceps*, the black-tailed phenotype was controlled by the recessive allele, so all black workers (rr) produce black-tailed (r) males. Only 2 *B. montivagus* workers ended up producing males, and each of them produced only 1 male offspring. Despite the limited sampling, one of the black-tailed workers produced a male with full orange in T5 (Fig. 6B). This suggests that orange is unlikely to be dominant and thus the inheritance of the locus is unlikely to be the same in *B. montivagus*. Together, this suggests that the evolution of mimetic color patterns of the Southeast Asia bumble bee species is driven by independent mutations.

## Discussion

### *Hox* Genomic Regions Are Genomic Hotspots for Color Pattern Evolution in Bumble Bees

It has been proposed that *Hox* genes may play a crucial role in color pattern formation and evolution in bumble bees (Rapti et al. 2014; Tian et al. 2019; Rahman et al. 2021), as these genes are responsible for segment-specific variation across animals and bumble bee color patterns vary more near *Hox*-transition boundaries along the body. The discovery of *cis*-regulatory mutations driving shifts in *Hox* gene expression (*Abd-B*) in *B. melanopygus* supported *Hox* genes not only being involved but holding the key mutations driving variation (Tian et al. 2019). Our results in *B. breviceps*, a distantly related species to *B. melanopygus* (∼22 million years divergence (Hines 2008)), also found the *Hox* region to harbor the mutations driving color morphs. This establishes *Hox* genomic regions as genomic hotspots regulating color pattern diversity in bumble bees.

Mutations in *Hox* gene regions can cause homeosis that is broadly believed to be nonadaptive, selected against and rarely seen in nature (Carroll 2005), and mostly only driving macroevolutionary changes (Pavlopoulos and Averof 2002; Gehring et al. 2009). Nevertheless, in both *B. melanopygus* and *B. breviceps*, the differential regulation of genes (either *Hox* genes themselves or *iab6* lncRNA) associated with *Hox* loci mutations occurs late in development (e.g. late pupal and early adult stages), when adult structures complete development. Consequently, shifts in *Hox* activity at this stage are able to mediate color phenotypes without causing large pleiotropic effects on other body structures. The use of pleiotropic, major effect loci as genomic hotspots of color radiation was also found in *Heliconius* butterflies (Reed et al. 2011; Martin et al. 2012; Nadeau 2016). Together, these data point to a more nuanced consideration of how developmental loci with strong pleiotropy are targeted in evolution.

On the other hand, although the *iab* region is targeted in color pattern evolution in both *B. breviceps* and *B. melanopygus*, the exact genomic locus and the regulatory effects of the mutation were different between the 2 species. While the mutation in *B. melanopygus* altered *Abd-B* expression, the *B. breviceps* locus is more likely to be driving differential expression of intergenic ncRNA, at least initially. This demonstrates the *iab* region can be modified in diverse ways to direct adaptive phenotypic evolution.

### The Orange/Black Color Shift May Be a Result of Homeosis

**Fig. 7.**
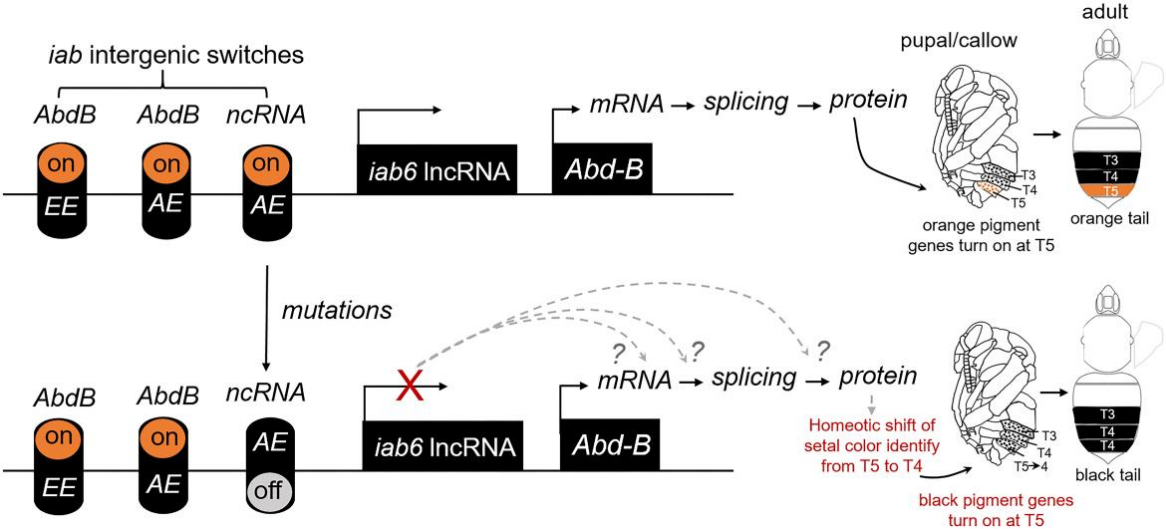
The proposed mode of genetic control of the orange/black color dimorphism in *B. breviceps.* The schematic diagram illustrates how mutations in the color locus may drive orange/black setal pigmentation changes. This model proposes that the color locus of *B. breviceps* may correspond to an *iab* genetic switch that controls *iab6* lncRNA expression in adult epidermis of metasomal T5. A mutation on the locus may cause the *iab6* lncRNA switch to turn off in the black-tailed form, which causes expression silencing of this lncRNA at T5. The loss of *iab6* lncRNA at the metasomal T5 may then interrupt the transcriptional or translational regulation of its target *Hox* genes, most likely *Abd-B*. Interruption of normal function of this *Hox* gene at T5 may then cause a homeotic shift of T5 setal identity toward the more anterior, black-colored T4 or T3, leading to the orange-to-black shift. Dashed arrows and question marks indicate untested alternative hypothesis on how ncRNA silencing may interrupt *Abd-B* during transcription and translation. EE, embryo epidermis; AE, adult epidermis.

The relative genomic position of the color locus of *B. breviceps* corresponds to the *iab-6*/7 region (Bender et al. 1983), a region known to regulate *Abd-B* gene expression to determine the identity of posterior para-segments (PS11-13) in embryos, which corresponds to the abdominal T5-6 (the posterior tail adjacent to the sting) in bumble bees (Lewis 1978; Regulski et al. 1985; Tian et al. 2019). Thus, the regulation domain of the color locus overlaps perfectly with the location of color dimorphism in *B. breviceps*, which also occur in abdominal T5. In *Drosophila*, laboratory-generated *iab-6*/7 mutants, which have a large part of the *iab-6* domain eliminated, show repressed *Abd-B* expression and display homeotic transformation of posterior segments into anterior ones (Lewis 1978; Regulski et al. 1985; Duncan 1987; Celniker et al. 1990). Similar to the fly mutant, the black-tailed bees have 2 genomic DNA deletions (3 and 46 bp deletion) on the color locus compared with the orange-tailed bees (Fig. 3A). While the expression of the *iab6* lncRNA clearly implicates this ncRNA, our data show signs, albeit not significant, of upregulation of *Abd-B* in the orange forms during setal pigmentation (P15 and QA; Fig. 5A). Thus, it is possible that *Abd-B* is also implicated in color variation in *B. breviceps* but with low-fold upregulation in the orange form. Therefore, a possible scenario for color shift is that in the black-tailed bees, the mutations on the color locus repress *iab6* lncRNA expression in the metasomal T5, resulting in *Abd-B* repression in this segment and consequently a homeotic transformation of identity of T5 from orange posterior segments to the identify of anterior segments (T3 or T4), which in *B. breviceps* bear black hairs (Fig. 7). This would suggest a potential homeotic shift in driving segmental variation, similar to what has been observed for *B. melanopygus* (Tian et al. 2019).

### The Function of the Intergenic lncRNA

The full length of *iab6* lncRNA remains to be characterized. Thus, the exact location of 3′ and 5′ ends remains unknown. The confirmed 383 bp fragment of the *iab6* lncRNA is 25 kb downstream *Abd-B* (Fig. 3B), is expressed only in the metasomal T5 (Fig. 5C and D), and is strongly associated with segmental phenotypes (Fig. 5C). The domain of this *iab6* lncRNA partially overlaps with the *iab8* lncRNA, a 90 kb, massive ncRNA spanning its domain from *iab-8* to *iab-2* and extensively characterized in *Drosophila* (Bae et al. 2002; Gummalla et al. 2012; Fig. 5B). It also shares partial domains with another identified lncRNA (known as the *iab-1* lncRNA) in the silkworm (Wang et al. 2019). However, the transcription direction of the *iab6* lncRNA is opposite to these previously identified ncRNAs, excluding the possibility that it is a subset of these known ncRNA species. Thus, the *iab6* lncRNA may present a novel intergenic lncRNA in the *iab* region.

How the *iab6* lncRNA regulates the orange black color shift remains unclear. Interestingly, none of the RNAi-treated, orange-tailed bees showed a complete color switch from orange to black on T5. Instead, all treated bees exhibited a mix of white and grayish back hairs on the T5 segment (supplementary fig. S4, Supplementary Material online). The fact that some orange hairs turned into white instead of black in response to *iab6* lncRNA silencing suggests that this lncRNA may be required for producing both orange and black colors, with perhaps relatively higher level of this lncRNA required for producing the orange color. Hines et al. (2017) demonstrate that the red- and black-colored bumble bee hairs differ primarily in relative proportion of phaeomelanin and eumelanin pigments. Therefore, the *iab6* lncRNA may exert dual function of activating melanin synthesis and fine tuning the proportion of different types of melanin in setal cells.

The downstream target of the *iab6* lncRNA leaves another question to be explored. Our gene expression analysis demonstrated segment-specific expression of the *iab6* lncRNA in the metasomal T5. This suggests that the key function of the *iab6* lncRNA is to maintain identity of this segment. Most known ncRNA transcribed from the *iab* regions are known to maintain segmental phenotypes via regulating *abd-A* and *Abd-B* expression (Ronshaugen et al. 2005; Akbari et al. 2006; Gummalla et al. 2012; Hui et al. 2013). The color locus and *iab6* lncRNA is physically closer to *Abd-B* relative to *abd-A*. In addition, the *iab6* lncRNA is specifically expressed in posterior abdominal segments, completely matching the expression domain of *Abd-B* (Bender et al. 1983; Brena et al. 2005). These all support its functional correlation with *Abd-B*. Nevertheless, our gene expression analysis revealed no significant difference in *abd-A* and *Abd-B* mRNA levels between orange- and black-tailed phenotypes. However, the orange-tailed bees do show a trend toward higher means in *Abd-B* expression during the same duration of the *iab6* lncRNA upregulation (Fig. 5A). Therefore, it is possible that the *iab6* lncRNA may serve to fine tune to expression of *Abd-B*. On the other hand, there are other possible ways where ncRNA can direct setal pigmentation through regulating *Hox* genes. For instance, some ncRNAs can direct alternative splicing of target mRNAs (Parikshak et al. 2016). Some cytoplasmic ncRNA can affect protein levels of target genes by regulating mRNA stability, degradation, and translation (Gong and Maquat 2011; Yoon et al. 2012; Thomson and Dinger 2016; Zhu et al. 2021). In addition, some ncRNAs have small open reading frames and encode micropeptides, which affect protein functions (Matsumoto et al. 2017). *Abd-B* or *abd-A* transcripts may also be regulated in earlier developmental stages (e.g. larval stage) than we examined here. Future studies characterizing the expression profiles at earlier developmental stages and examining splicing variants, protein expression, and protein activity of *abd-A* and *Abd-B* would facilitate better understanding of whether and how the *iab6* lncRNA and *Hox* genes interact to direct setal pigmentation.

Our study adds to recent discoveries of roles of lncRNA in driving adaptive divergence (Luo et al. 2019; Zimmer-Bensch 2019; Zhu et al. 2021; Du et al. 2023; Li et al. 2023; Monniaux 2023). The discovery of this novel lncRNA in the *Hox* region is of particular significance as it helps in understanding how these pleiotropic developmental loci can be regulated to drive rapid phenotypic evolution.

### Lack of Conservation in Color Locus Underlying Mimetic Color Pattern Convergence

Although this study and Tian et al. (2019) demonstrate the *Hox iab* region is a genomic hotspot for bumble bee color pattern evolution, both studies also support convergent color variation of comimics resulting from different sets of mutations. Although deletions have been implicated here for *B. breviceps*, these same deletions do not affect orange tail color in comimics, supporting potential for multiple mutations to alter this phenotype by targeting the same regulatory locus. Using diverse ways of targeting the same locus was also found in *Heliconius*, where comimetic *Heliconius melpomene* and *Heliconius erato* come about develop the same color patterns by targeting the same genes but in different regulatory modules of those genes (Joron et al. 2006; Baxter et al. 2008; Papa et al. 2008; Martin et al. 2012). Overall, this supports the role of novel mutations in phenotypic convergence, but it also suggests that certain loci are optimal targets for generating change.

## Conclusion and Future Perspectives

Our study, combined with previous research on *B. melanopygus*, supports genomic regions of *Hox* genes as genomic hotspots for driving color pattern radiation in *Bombus*. The repeated involvement of the *iab* region in color pattern evolution by different species, through mutations on different genomic loci, and the discrepancy between *B. breviceps* and *B. melanopygus* on the effects of their color locus on gene regulation demonstrate the remarkable evolvability of *Hox* loci and the diverse ways by which these highly pleiotropic loci can drive rapid phenotypic radiation. These data also support that altering gene expression in a tissue- and stage-specific manner is likely a common way by which highly pleiotropic regulatory loci like *Hox* loci can drive evolution of novel adaptive traits while minimizing pleiotropic impacts. So far, understanding of functions of the *Hox* genes and their intergenic regions is largely restricted to a few model organisms. Most of these studies tend to emphasize the functional and structural conservation of *Hox* across animals and plants or its role in directing macroevolutionary changes between higher taxa. Studies on bumble bee color genetics, however, demonstrate that the *Hox* loci can undergo rapid evolutionary changes to inform microevolutionary processes. Future studies on this group focusing on functional validation of the color locus, including using CRISPR or further RNAi approaches, and studies on mechanisms by which lncRNA regulate downstream targets would expand our understanding on the diversity and function of the *iab* region as well as *iab* ncRNAs and provide more insights into how *Hox* clusters operate in nature to direct adaptive phenotypic evolution.

## Methods

### Identifying the Color Pattern Transition Zone

Previous literature records support the orange tail phenotypes being broadly distributed in the species’ western ranges, including the Himalaya regions, highland southwestern China, and the adjoining higher mountainous areas of Myanmar, Laos, and Thailand, while the black-tailed phenotypes occur in a restricted area of the eastern Himalaya and the broad mountainous area of southeastern China and northern Vietnam (Hines and Williams 2012). Thus, the transition zones for this morph include the eastern Himalaya and southern China. We performed detailed analysis on color pattern distribution in southern China, by documenting color patterns of specimens collected from across this region. *Bombus breviceps* samples used for color pattern analysis include those that are field collected (*n* = 631) during 2020–2022 from southern China and museum species. Color patterns were characterized following the methods described by Williams (Williams 2007). The dorsum of the bee was divided into 24 elements in females and 26 elements in males. Hair color was scored separately for each element, with each element assigned the color that occurred over 50% of the area. The minority color was also coded when it formed strongly contrasting fringes or spots. For each collection site, the ratio of the 2 color forms was calculated.

### Bee Collection and Rearing

Posthibernating queens of the orange- and black-tailed phenotype were collected from the transition zone (Fig. 2) in Guizhou. The queens were reared separately in plastic nest boxes and provided with fresh honeybee-collected willow pollen and 60% sugar water (sucrose:fructose:water = 1:1:2). They were allowed to established colonies at 29 ± 1 °C and 60 ± 5% relative humidity in complete darkness. All workers from the lab-reared colonies were preserved. Because workers can lay unfertilized eggs and produce male offspring when isolated from their maternal colony, we also establish microcolonies led by orphaned workers. For each colony, we prepared 8–16 microcolonies, each containing 3–5 workers bearing the same phenotype on metasomal T5, and the progeny males were preserved. Phenotypes on metasomal T5 (either orange or black) of workers, queen-produced males, and males produced from worker-led microcolonies were documented.

### GWAS

To reveal the loci controlling the orange and black on the last abdominal segment of *B. breviceps*, 44 laboratory breeding males (progeny of wild-caught queen from the color transition zone; supplementary table S3, Supplementary Material online) were selected for GWAS, including 22 orange and 22 black. All males selected for GWAS show no difference on color of other body regions (e.g. only the first metasomal segment yellow; Figs. 2 and 3) but discrete differences on metasomal T5. Among 44 selected males, with 22 males of each phenotype, 16 pairs of orange and black males were derived from 12 heterozygous (Rr) workers (e.g. 1 orange- and 1 black-tailed male from each worker), 6 orange-tailed males were derived from homozygous RR orange workers, and 6 black-tailed males were derived only from black-tailed (rr) workers. Males are haploid, thus using males for GWAS avoids heterozygosity and dominance effects. Total genomic DNA was extracted from the thorax muscle tissue using Qiagen DNeasy DNA kit, following manufacturer's protocol. Insert size libraries of 300–600 bp were constructed at Berry Genomics Corporation (Beijing, China). DNA libraries were sequenced on Illumina NovaSeq 6000 platform with 150 bp paired-end (PE) reads. Raw sequence data were filtered and trimmed using fastp v0.21.0 for quality control. Around 5 Gb of clean data were obtained for each sample (target raw depth around 20×; supplementary table S4, Supplementary Material online). Reads were then mapped to the published chromosomal-level genome of *B. breviceps* (Sun et al. 2020) using Burrows–Wheeler Alignment (BWA) mem (Li and Durbin 2009). Multisample variant calling and generating of the joint SNP data set and hard filter were performed using Genome Analysis Toolkit (GATK) v4.0.4 (McKenna et al. 2010). Using PLINK, reliable candidate SNPs that matched minor allele frequency (maf) > 0.05 and missing rates <0.2 were retained and transcoded into tped format. The kinship matrix of samples was calculated first, and then, genotype–phenotype association analysis between case-control phenotypes (orange and black) and the SNP data set was performed in Emmax (Kang et al. 2010) based on a mixed linear model. The GWAS results were visualized using a Manhattan plot and QQ plot based on CMplot in R.

### Genotyping

To narrow the locus regulating the phenotype, genotyping was performed on 41 orange and 51 black workers (supplementary table S5, Supplementary Material online). Samples used for narrowing the locus primarily included workers wild-caught from the phenotypic transition zone in Guizhou and Yunnan but also included those from areas where exclusively orange or black morphotypes occurred in Sichuan, Guangdong, Guangxi, Fujian, and Zhejiang. Individuals were subsampled for SNPs and indels across the fixed 17.6 kb interval to look for potential recombinants, which were then sequenced across all SNPs and indels to better define the window of fixation. DNA was extracted with Qiagen DNeasy or Tiangen DNA kits, purified with NanoDrop 2000 (Thermo Scientific) and PCR amplified using EmeraldAmp® MAX PCR Master Mix kit (Takara) with custom-designed primers (supplementary table S6, Supplementary Material online). Sanger sequencing was performed at the Tsingke Biotechnology Co., Ltd (China). SNPs were called from chromatograms with heterozygosity determined from double peaks. Finally, all SNPs and indels in the narrowed fixed interval were sequenced (supplementary table S5, Supplementary Material online) for 16 individuals of other geographic populations, including Himalaya, Myanmar, and Thailand to test the if there is a population-wide genotype association of this locus with color phenotypes, for 2 *B. grahami* workers to test color pattern evolution history, and for different morphs of *B. montivagus* (4 orange and 3 black worker) and *B. haemorrhoidalis* (6 black-tailed worker only, as no orange-tailed sample was collected during the time this research was performed) to test if this locus is associated with parallel phenotypes of these comimics. Primers used for the genotyping the fixed color locus is provided in supplementary file, Supplementary Material online (supplementary table S7, Supplementary Material online).

### Phylogenetic Tree Construction and Haplotype Analysis of Color Locus

Haplotype phasing and haplotype network analysis were performed using Network 10.2.0. software. Maximum likelihood (ML) trees of phased haplotypes for the color locus were constructed in MEGA11 (Tamura et al. 2021) based on the JC69 model.

### RNA Extraction and cDNA Preparation

Epidermal tissues of the abdominal tergite segment were dissected from worker pupae and adults in ice cold 1× phosphate-buffered saline (PBS) buffer, with fat body and muscle tissues removed. Tissue samples were flash frozen on dry ice immediately after dissection and stored at −80 °C. Each tissue sample was dissected and collected quickly (e.g. within 5 min) to prevent RNA degradation. RNA was extracted using the RNeasy® Micro Kit (Qiagen). RNA extractions, purification, and subsequent DNase treatment were performed following manufacturer recommendations, with purity and concentration of RNA confirmed using a NanoDrop 2000 (Thermo Scientific). For transcriptome sequencing, RNA was quantified by Qubit 3.0 with Qubit RNA Broad Range Assay kit (supplementary data, Supplementary Material online). For subsequent qPCR and PCR experiments, first-strand cDNA was synthesized from 500 ng of total RNA using the PrimeScript™ RT reagent Kit (Perfect Real Time) with gDNA Eraser Kit (Takara).

### Transcriptomics

Two micrograms of total RNA isolated from the T5 epidermal tissues of QA stage workers was used for stranded RNA sequencing library preparation. mRNA libraries were prepared using KC-Digital Stranded mRNA Library Prep Kit for Illumina® (Wuhan SeqHealth Co) following the manufacturer's instruction. The library products corresponding to 200–500 bps were enriched, quantified, and finally sequenced on Illumina NovaSeq 6000. Multiplexed libraries were sequenced at 150 bp PE using the Illumina HiSeq at the KangCe sequencing company (Wuhan, China). Raw sequencing data were first filtered by Trimmomatic (version 0.36) (Bolger et al. 2014). Low-quality reads were discarded, and adaptor sequences were trimmed. Clean reads were further treated with in-house scripts to eliminate duplication bias introduced in library preparation and sequencing (Shugay et al. 2014; Ma et al. 2018). For each color form, 3 workers (supplementary table S8, Supplementary Material online) were dissected and tissue samples were pooled for transcriptome sequencing. Deduplicated consensus sequences mapped to the reference genome of *B. breviceps* (Sun et al. 2020) using STAR software (version 2.5.3a) (Dobin et al. 2013) with default parameters. Read coverage (Fig. 5C) on the genome was visualized using IGV software (https://igv.org/). We specifically focused on reads corresponding to the *iab* region to look for RNAs transcribed from this region. Summary statistics for transcriptome sequencing was provided in supplementary table S9, Supplementary Material online. The detected ncRNA reads were further validated by Sanger sequencing with custom-designed primers (supplementary table S10, Supplementary Material online).

### Quantitative Real-Time PCR

To assess expression patterns of *abd-A* and *Abd-B*, and the *iab6* lncRNA, qRT-PCR was performed for epidermal tissues of dimorphic T5 at 12 developmental stages (Tian and Hines 2018) of both orange- and black-tailed phenotypes, including 5 pupal stages (e.g. P4, P10, P12, P13, P15), 4 pre-eclosion adult stages (e.g. 0, 3, 6, 12 h QA), and 3 callow stages (0, 6, and 12 h callow). We also collected epidermal tissues of the nondimorphic metasomal T3 for workers at stage P4, P10, P13, QA, and 0 h and 12 h callow for both phenotypes to examine expression of *iab6* lncRNA. Epidermal tissue dissection and RNA extraction were performed following the same method described above. qRT-PCR was performed in triplicate using TB Green® Premix Ex Taq™ II (Tli RNaseH Plus) Kit (Takara) on an CFX96 real-time PCR detection system. The housekeeping gene *v-ATPase*, which has previously been validated to be stably expressed during development (Tian et al. 2019) was used as reference gene. A standard curve (5× dilution series) was used to quantify gene expression. The expression level of target genes was normalized by *v-ATPase* (target gene/*v-ATPase*). Three replicates were used for each developmental stage. Two-sample *t*-test was performed to test differences of target gene expression level between color forms at each developmental stage. Primer sequences for target genes are provided in supplementary table S11, Supplementary Material online. Raw CT value generated by qRT-PCR is available in the supplementary data, Supplementary Material online.

## Supplementary Material

msad261_Supplementary_Data

## Data Availability

For both GWAS and transcriptome analysis, sequencing data are deposited in the Genome Sequence Archive (GSA) database under the BioProject #PRJCA018806 (accession number: CRA012106 for genome resequencing data; CRA012107 for transcriptome data). In addition, sequences of the fixed color locus generated by Sanger sequencing as well as those generated by haplotype phasing are provided in the supplementary data, Supplementary Material online (supplementary data, Supplementary Material online) and also uploaded to the treebase (http://purl.org/phylo/treebase/phylows/study/TB2:S30905).
