# Supplementary material for: Genetic Modification of a Hox Locus Drives Mimetic Color Pattern Variation in a Highly Polymorphic Bumble Bee
Source: Mol Biol Evol. 2023 Dec 1;40(12):msad261. doi: 10.1093/molbev/msad261 (PMC10724181; doi:10.1093/molbev/msad261)
Supplement: msad261_Supplementary_Data [file msad261_supplementary_data.zip › [4]_MBE-23-0743_Revision-supplementary_information-final.pdf]

Genetic modification of a *Hox* locus drives mimetic color pattern variation in a highly polymorphic bumble bee

Wanhu Yang[1], Jixiang Cui[1], Yuxin Chen[1], Chao Wang[1], Yuanzhi Yin[1], Wei Zhang[2] Shanlin Liu[1], Cheng Sun[3], Hu Li[1], Yuange Duan[1], Fan Song[1], Wanzhi Cai[1], Heather M Hines[4], Li Tian[1*]

[1]Department of Entomology and MOA Key Lab of Pest Monitoring and Green Management, College of Plant Protection, China Agricultural University, Beijing 100193, China
[2]State Key Laboratory of Protein and Plant Gene Research, School of Life Sciences, Peking University, Beijing 100871, China
[3]College of Life Sciences, Capital Normal University, Beijing 100048, China
[4]Department of Biology, the Pennsylvania State University, 208 Mueller Laboratory, University Park, PA, 16802, USA

*Correspondence: Li Tian
E-mail: ltian@cau.edu.cn

Supplementary Information

This PDF file includes:

Supplementary results
Supplementary methods
Supplementary figures S1 to S4
Supplementary tables S1 to S11
Supplementary references

Supplementary results

Phenotypic transition zone We performed detailed analysis on color pattern distribution in south China, by documenting color patterns of specimens newly collected across this region. We identified a broad phenotypic transition zone between the orange and black-tailed phenotypes in the southeast edge of the Yunnan-Guizhou plateau (fig. 2). This transition zone is approximately 300km in width from northwest to southeast and 600km in length from northeast to southwest, involving southern Yunnan, all of Guizhou, and southern Hunan. This transition zone occurs across altitudinal gradients, with the orange/black ratio gradually decreasing from northwest to southeast, largely concordant with the topographic transition from the Yun-Gui highlands to the lower elevation mountainous area of southeast China (fig. 2).

The genetic inheritance of orange/black phenotypes We first documented phenotypic segregation among workers of *B. breviceps* colonies headed by wild-caught queens. We collected post-hibernation orange and black tailed queens from the phenotypic transition zone, where it would be more likely to obtain heterozygous genotypes for estimating inheritance. From the 60 queens collected, 39 successfully established colonies, including 29 orange and 10 black tailed queens. Within the female progeny no caste or size related color variation was found, as has been noted with some bumble bee species. Colonies produced either orange or black tailed individuals with no intermediates detected, suggesting a dimorphic phenotype.

Color ratios among the male and female progeny, as well as the inferred mating history of the wild-caught queens, are summarized in Table S1. Colonies headed by orange-tailed queens have all-orange workers or a fairly even (50:50) mixture of orange and black workers. Colonies headed by black queen produced either all-orange or all-black workers. Orange tailed queens can produce either all orange males or a mixture of orange and black males, whereas black queens can only produce black males. Given the haplodiploid sex determination system in bumble bees (males are haploid offspring of unfertilized diploid female eggs), this supports the color being regulated by a single locus, with orange dominant to black. For example, an orange queen producing both orange and black worker and males must be heterozygous (R/r) and mated with a black male (r), and the expected color ratio among workers (e.g., orange R/r : black r/r) and those among males (e.g., orange R : black r) should all be close to 1:1. These expected color ratios were met in all colonies led by orange queen and sporting admixed phenotypes (supplementary table. S1). Because bumble bee worker can lay eggs and produce haploid males, we also investigated phenotypic segregation among worker derived male progeny to further confirm the inferred inheritance. We established

microcolonies comprising 3-5 orphaned workers with the same phenotype. Orphaned bumble bee workers would quickly establish a dominancy hierarchy, resulting in single dominant worker producing offspring. Assuming only one worker per microcolony produced offspring (Owen & Plowright, 1980; Princen et al, 2020; Ge et al, 2021) an orange tailed worker would produce either all orange males (if the worker is homozygous) or a mixture of orange and black males with a phenotypic ratio of 1:1 (if the worker is heterozygous), whereas a black worker should produce only black males. Color ratios among worker-derived males in all of our micro-colonies exhibited the expected segregation ratios under the single-locus, orange-dominant hypothesis (supplementary table. S1).

To further validate the single-locus model, we performed controlled crossing among the progeny queen and males and documented the phenotypic segregation of F1 worker and queen-produced males. Orange queens were crossed with black and orange males, resulting in four mating types: RR X R, RR X r, Rr X R, Rr X r, Black queens were also crossed with orange and black males, resulting in two mating types: rr X R, rr X r. (supplementary fig. S2). All of the established colonies produced a phenotypic ratio that is consistent with the single-locus model (supplementary fig. S2 and table S2). Therefore, our rearing results strongly suggest that the orange and black color switch on metasomal T5 in *B. breviceps* is controlled by a single, biallelic Mendelian gene, with the orange (R) allele dominant to the black (r).

The evolutionary history of orange-black dimorphism in *B. breviceps* Our genetic analysis of the color locus of *B. breviceps* along with its orange-tailed sister species *B. grahami*, suggests that the orange coloration is the ancestral functional phenotype and that the black tails is a mutated variant from this. This hypothesis, however, requires further study as some of the black tailed lineages of *B. breviceps* and its close relatives were not involved in our genotyping assay. For instance, *B. genalis*, the sister species to the *B. breviceps*+*B. grahami* clade, sport all black colors throughout its body, including metasomal T5 (Williams, 2022). Black tailed phenotypes also occur in the black-thorax/white pleuron lineages in eastern Himalayan regions (Williams, 2022). Studying these could reveal whether similar segmental phenotypes may be convergently evolved or resulted from ancestral allele sorting (Hines et al, 2011).

Supplementary methods

Color pattern distribution analysis *B. breviceps* samples used for color pattern analysis include those that are filed collected between 2020-2022 form south China and museum species (supplementary data). We used 631 filed collected and 620 museum

specimens for color pattern documentation. Color patterns were characterized following the methods described by Williams (2007). In general, the dorsum of the bee was divided into 24 elements in females and 26 elements in males. Hair color was scored separately for each element. The color patterns were coded as pubescence color, which covers over 50% of the element area. The minority color was also coded when it formed strongly contrasting fringes or spots. Percentage of individuals sporting the orange and black T5 was calculated.

Color pattern segregation among progeny of wild-caught queens Post-hibernating queens of the orange and black tailed phenotype were collected from the transition zone in Guizhou. The queens were reared separately in plastic nest boxes and provided with fresh honeybee collected willow pollen and 60% sugar water (sucrose: fructose: water = 1:1:2). They were allowed to established colonies at 29±1°C and 60 ± 5% relative humidity in complete darkness. All workers and queen produced males (males produced from the colony before queen death) from the lab reared *B. breviceps* colonies were preserved. We also isolated workers from the colony to establish micro-colonies. For each colony, 8-16 micro-colonies, containing 3-5 workers bearing the same phenotype on metasomal T5 were set up and the progeny males were preserved. Phenotypes on metasomal T5 (either orange or black) of workers, queen produced males and males produced from worker-led micro-colonies were documented. The proportion of orange and black phenotypes among these progenies were calculated following previously described methods.

Color pattern segregation among progeny of controlled crossing Young virgin queens and males reared from the colonies were subsequently mated in flight cages. New queens and males of specific phenotypes and genotypes (speculated from its maternal colony) were crossed. Orange and black tailed queens were crossed with black or orange tailed males, producing several crossing scenarios (e.g., RR×R, RR×r, Rr×r, rr×R, rr×r, Rr×R) (supplementary fig. S2). Mating was performed in a 30cm×30cm×30cm mesh box under full spectrum plant growth light. Successfully mated queens were kept in a darkroom for 3-5 days at an ambient temperature of 29±1°C and a relative humidity of 60%, fed with fresh bee pollen and sugar solution, after which the queens were stored at 4°C for 3 months in 20ml plastic vials half filled with a moist tissue paper. The queens were then resuscitated and installed in nest boxes to obtain a second generation of colonies. F1 worker and queen produced F1 males were preserved and their phenotypes on metasomal T5 were subsequently documented.

RNA interference RNA interference (RNAi) was performed for *iab6* lncRNA through injection of small-interfering RNA (siRNA) targeting *iab6* lncRNA (si-*iab6lncRNA*). This was designed based on partial sequence of the *iab6* lncRNA and synthesized by Guangzhou RiboBio Co., Ltd. (Guangzhou, China). 30ug si-*iab6 lncRNA* were injected into individual P14-stage worker pupae, using a microinjector (Nanoliter 2000 Injector; WPI Inc., Sarasota, FL, USA). After injection, pupae were allowed to continue developing into the QA stage in an incubator (30℃; Relative humidity=50%, complete darkness). To validate the efficiency of RNAi, injected individuals were dissected at the QA stage, a point where external pupal cuticle has already been shed. RNA was extracted from the epidermal tissue of the fifth abdominal tergite. Quantitative real-time PCR was performed as outlined in the Methods to examine the relative expression level of *iab6* lncRNA. Individuals without injection (Non-injection) and injected with control siRNA (nc-siRNA) were used as negative controls. Three biological replicates (e.g. three worker bees) were performed for each control and treatment. A Shapiro-Wilks test was used to validate normality of qRT-PCR data. One-way Analysis of Variance (One-way ANOVA) and Turkey's HSD post hoc test was performed to test differences among treatment groups.

To examine the phenotypic impact of *iab6* lncRNA knockdown, worker bees of both treatment and control groups were collected at the QA stage and reared for another 24 hours to allow hair color to continue to develop fully (Tian and Hines, 2018). During this time period, bees were individually reared in plastic containers in the incubator, and were fed with 60% (g/ml) sugar solution. After 24-hours, these bees were killed by freezing at -20 ℃ for 20 minutes and their hair color on metasomal T5 was examined under a stereomicroscope. To further examine more detailed color change within hairs, individual hairs were examined using light microscope (Nikon, SMZ1) under higher magnifications (~200Ｘ).

Supplementary figures

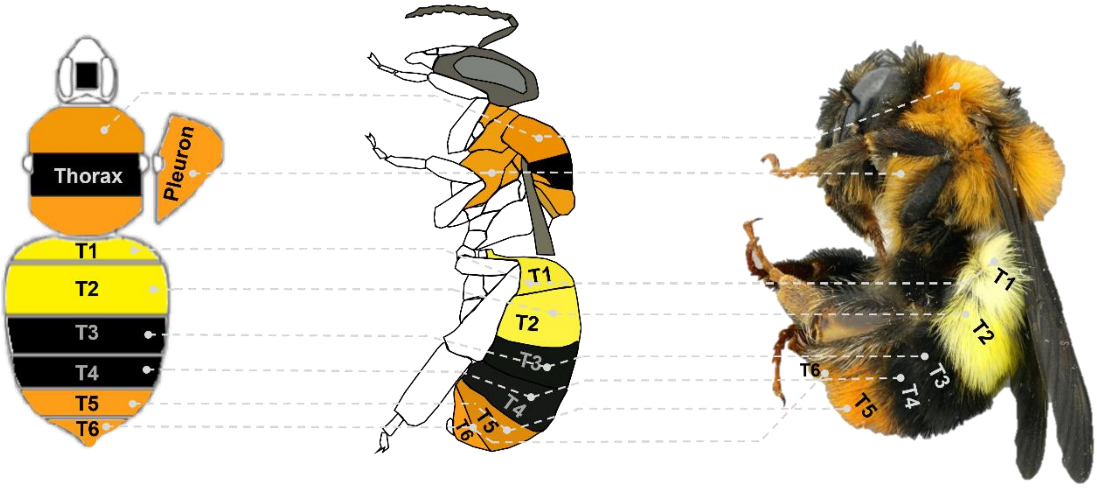

Fig. S1. Body segments of full-developed adult *B. breviceps* worker bee. Diagrams on the left and middle indicate dorsal and side view of a worker bee. The image on the right represents a side views of an adult worker bee. Thorax, pleuron and abdominal tergal plate (T1-6) are illustrated. The same body part of the three images are connected with dash lines.

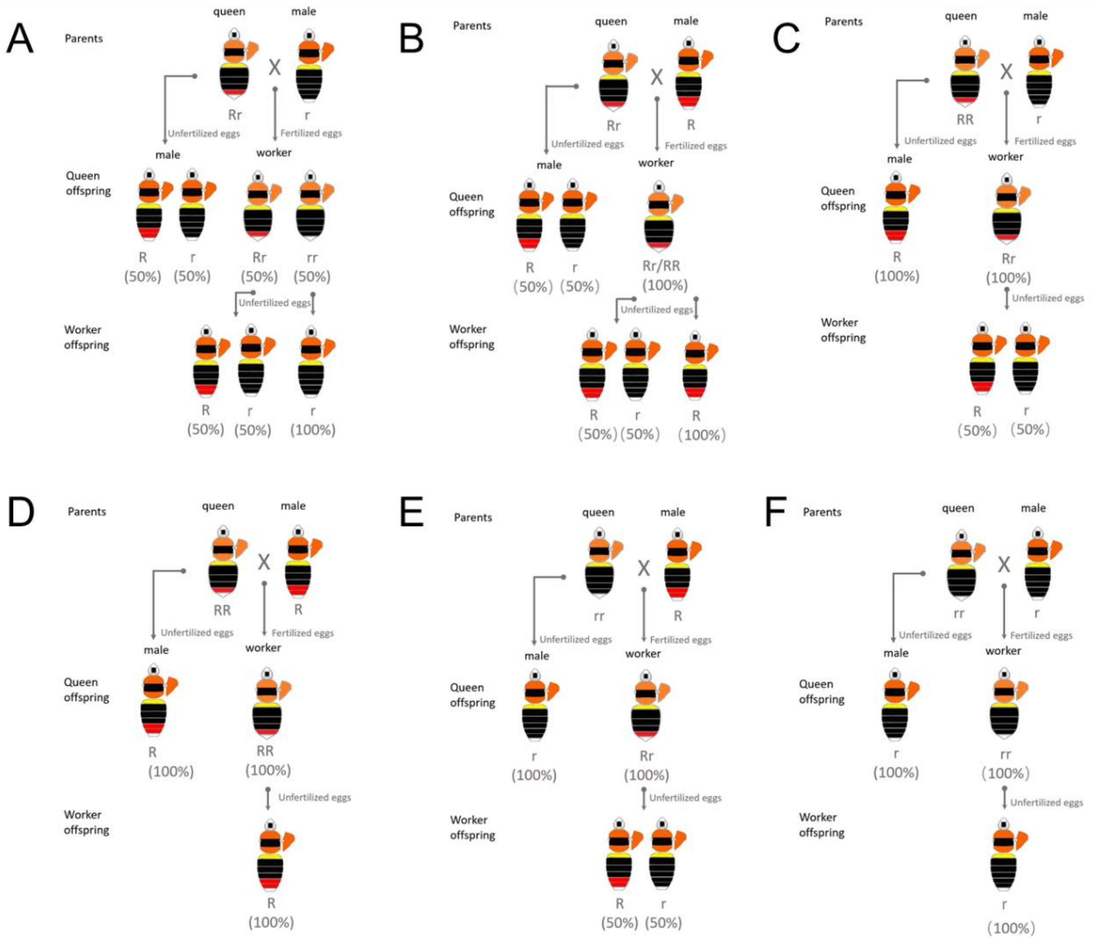

Fig. S2. Schematic diagram of controlled genetic crosses for *B. breviceps*. A-F illustrate six crossing scenarios performed between color forms. The letter “R” and “r” below each color pattern graph indicates inferred genotypes based on the single locus inheritance model, with R and r representing the dominant and recessive allele, respectively. Virgin queens and males used for genetic crossing were derived from colonies established by wild collected queen. Their genotypes were inferred from color form of their mother queen, phenotypic segregation among other offspring of their maternal colonies as well as their own color forms, based on the single-locus hypothesis. Numbers in the parentheses indicates hypothetical percentage of orange/black tailed color form among F1 offsprings of the queen, as well as among male offspring derived from the F1workers, under the single locus inheritance hypothesis. In all crossing scenarios, phenotypic segregation among offspring supports the orange/black dimorphism on metasomal T5 being controlled by a single Mendalian locus, with the orange allele dominant over black allele.

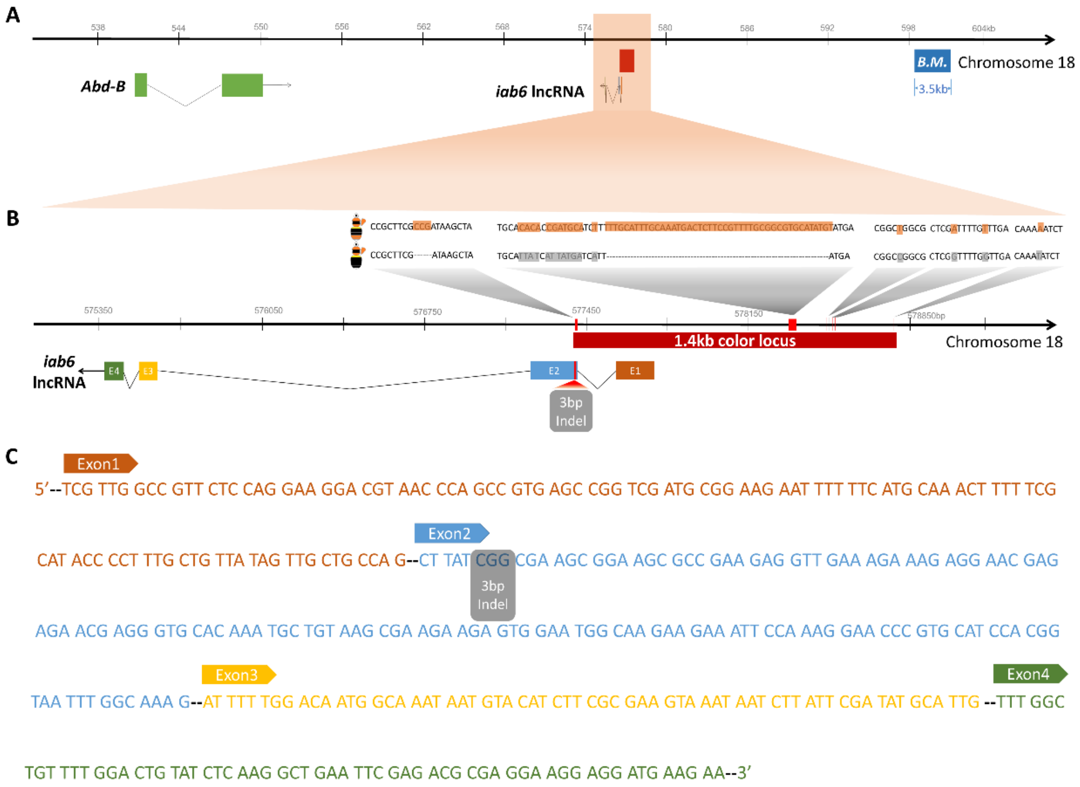

Fig. S3. Position, structure and sequence of the *B. breviceps iab6* lncRNA. A. The relative position of *Abd-B* gene, color loci of *B. breviceps* and *B. melanopygus* (B.M.) and the *iab6*-lncRNA on the *B. breviceps* genome (chromosomal 18). Note that the color loci of the two species are ~25 kb apart from each other. B. A zoom-in view of genomic region surrounding the 1.4-kb color locus showing the relative position and structure of the *iab6* lncRNA transcripts. The red bar indicates the 1.4-kb color locus. The sequence above the red bar indicates partial sequence of the color locus in the orange and black tailed bee, with indels and SNPs highlighted in orange (orange tailed bee) and gray (black tailed bee). Below the red bar, the position and structure of the *iab6* lncRNA was illustrated, with the four exons (E1-E4) indicated by different colored boxes. Note that this lncRNA partially overlaps with the color locus, spanning the 3-bp indels, resulting in the transcript of the black-tailed bee being 3 bp shorter than that of the orange-tailed form. C. The partial sequence of *iab6* lncRNA expressed in the orange-tailed form. Different colors indicate the four exons the *iab6* lncRNA contains. The missing 3-bp (indel) in the black form is highlighted in grey.

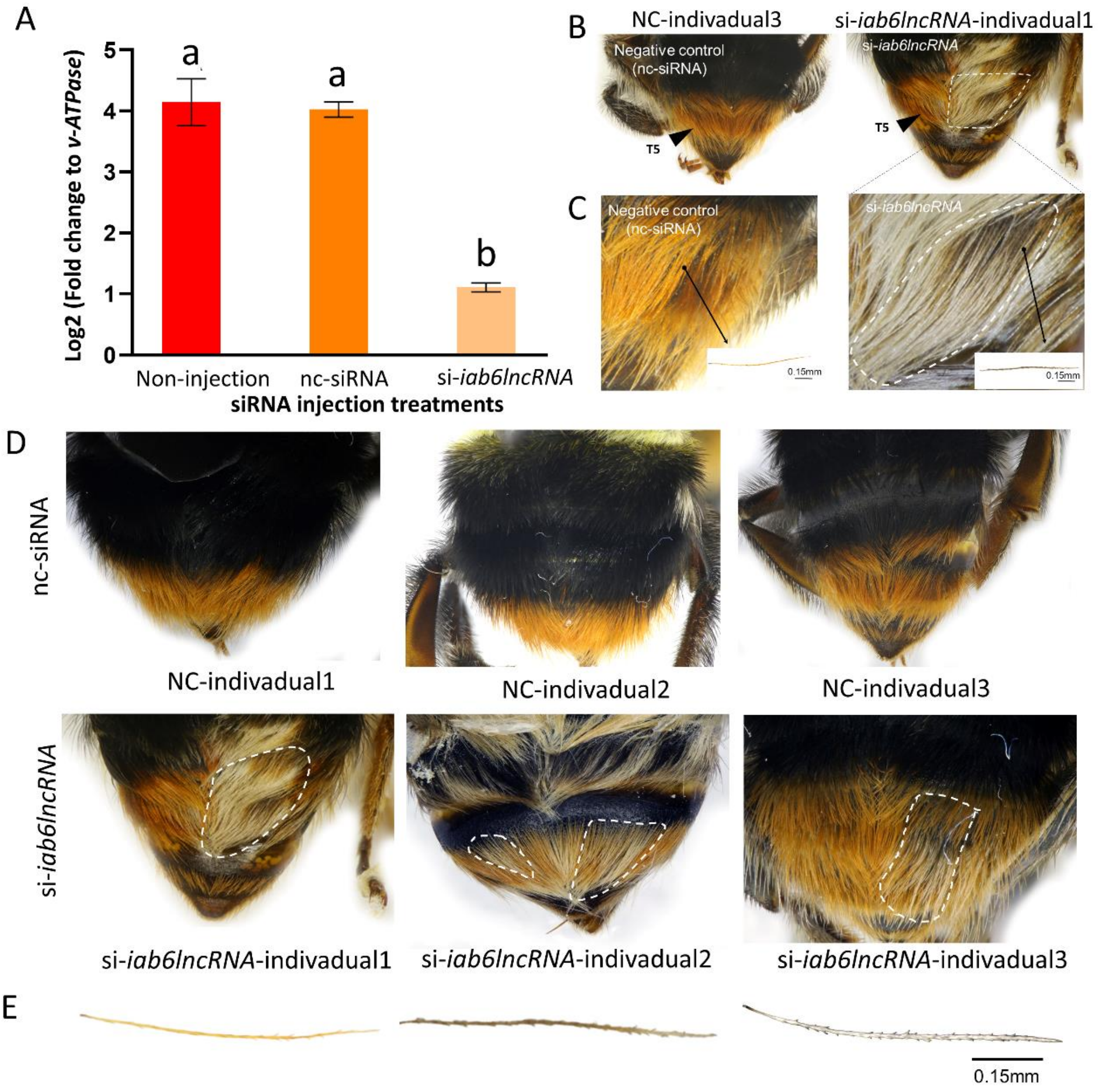

Fig. S4. Phenotypic impacts of *iab6* lncRNA knockdown A. *iab6* lncRNA expression level at the QA stage in control (Non-injection and nc-siRNA) and treatment (si-*iab6lncRNA*) groups. Error bars indicate standard error (SE) of the mean. Means with different letters are significantly different from each other (One-way ANOVA followed by Turkey's HSD posthoc test, $P<0.05$; N=3 for all groups). Note that injection of *si-iab6lncRNA* at P14 pupal stage caused a ~8 fold knockdown of the *iab6* lncRNA at the QA stage. B-E. Effects of *iab6* lncRNA knockdown on hair color of abdominal T5 of orange-tailed bees. White dashed circle indicates the affected area. Images in (B) represent a broad dorsal view of T5 in 24-hour callow workers. Note that *si-iab6lncRNA* injected group show part of the T5 turning grey, while the control group (nc-siRNA) remains fully orange. C. Close up view of the affected area of T5. The affected areas are highlighted by white dashed lines. Small images in the white boxes on the left corner of control and treatment group are close up view of single hairs. Note that in the *si-*

iab6lncRNA injected group, some hairs on the T5, which would otherwise turn orange at 24 hour callow, become white or greyish black at the base of the hair. Images in (D) show the phenotypes of T5 of all three individuals of negative control and *si-iab6lncRNA* treatments, with affected area circled by white dashed lines. Images in (E) show single hair of T5, including, from left to right, an orange hair from the untreated individual, a greyish black hair pulled off from the affected area of T5 of a RNAi treated worker bee, and a white hair pulled off from the affected area of T5 of a RNAi treated worker bee.

Supplementary tables

Table S1. Phenotypic segregation among progeny of wild collected *B. breviceps queens* and male progeny of worker-led micro-colonies

Colony	Queen		Worker			Worker produced male					
	Queen color	Inferred mating	Orange	Black	$\chi^2(1:1)$[a]	Worker color	Inferred worker genetype	N[b]	Orange	Black	$\chi^2(1:1)$
TL25-14	Orange	RR X R	237	0		Orange	RR	4	26	0	
TL25-43	Orange	RR X R	215	0		Orange	RR	4	7	0	
TL27-14	Orange	RR X R	132	0		Orange	RR	5	14	0	
TL27-17	Orange	RR X R	119	0		Orange	RR	3	3	0	
TL27-70	Orange	RR X R	172	0		Orange	RR	5	14	0	
TL27-72	Orange	RR X R	142	0		Orange	RR	3	1	0	
TL25-12	Orange	Rr X R	102	0		Orange	RR/Rr	5	4	0	
TL27-41	Orange	Rr X R	186	0		Orange	RR/Rr	7	24	0	
TL27-64	Orange	RR X R	203	0		Orange	Rr	3	2	2	0
TL25-60	Black	rr X R	119	0		Orange	Rr	7	9	15	1.500
TL27-36	Black	rr X R	209	0		Orange	Rr	7	16	19	0.257
TL27-48	Black	rr X R	222	0		Orange	Rr	7	16	16	0
TL27-74	Black	rr X R	311	0		Orange	Rr	14	35	41	0.474
TL25-47	Orange	Rr X r	71	56	1.772	Orange	Rr	7	16	15	0.032
						Black	rr	7	0	41	
TL25-48	Orange	Rr X r	99	116	1.344	Orange	Rr	7	10	10	0
						Black	rr	4	0	16	
TL27-1	Orange	Rr X r	107	105	0.019	Orange	Rr	6	8	10	0.222
						Black	rr	6	0	12	
TL27-13	Orange	Rr X r	147	121	2.522	Orange	Rr	5	3	4	0.143
						Black	rr	3	0	7	
TL27-44	Orange	Rr X r	86	111	3.173	Orange	Rr	3	2	3	0.200
						Black	rr	3	0	2	
TL25-59	Black	rr X r	0	181		Black	rr	7	0	15	
TL27-40	Black	rr X r	0	230		Black	rr	9	0	42	
TL27-59	Black	rr X r	0	172		Black	rr	8	0	25	

[a] $\chi^2(1:1)>3.84$ means the ratio is significantly different from 1:1

[b] N means number of worker micro-colonies used

Table S2. Phenotypic segregation among progeny from controlled genetic crossing

Colony	Parents						F1 worker color		
	Queen			Male					
	Origin[a]	Color	Genotype[b]	Origin	Color	Genotype	Orange	Black	$\chi^2(1:1)$[c]
y22-29	TL27-64	Orange	RR	TL27-1	Orange	R	135	0	
y22-49	TL32-7	Orange	RR	TL27-1	Orange	R	221	0	
y22-35	CYX5-2	Orange	RR	TL27-74	Black	r	124	0	
y22-2	TL25-45	Orange	Rr	TL27-74	Black	r	57	61	0.136
y22-13	CYX5-2	Orange	RR	TL27-74	Black	r	94	0	
y22-38	CYX5-2	Orange	RR	TL27-36	Orange	R	24	0	
y22-30	TL27-41	Orange	Rr	TL27-74	Black	r	42	38	0.200
y22-50	TL27-13	Orange	RR	TL25-59	Black	r	73	0	
y22-6	CYX5-2	Orange	RR	TL27-1	Orange	R	158	0	
y22-28	TL32-1	Orange	RR	TL27-1	Orange	R	93	0	
y22-25	TL27-36	Orange	Rr	TL27-36	Black	r	26	21	0.532
y22-54	TL27-64	Orange	RR	TL27-1	Orange	R	23	0	
y22-57	TL32-1	Orange	RR	TL27-1	Orange	R	15	0	
y22-58	CYX5-2	Orange	RR	TL27-1	Orange	R	164	0	
y22-36	TL27-1	Orange	Rr	TL27-74	Black	r	75	69	0.250
y22-51	CYX5-2	Orange	RR	TL27-1	Orange	R	56	0	
y22-44	TL32-1	Orange	RR	TL27-1	Orange	R	29	0	
y22-48	TL27-36	Orange	Rr	TL25-14	Orange	R	32	0	
y22-19	CYX11-2	Black	rr	CYX4-5	Orange	R	125	0	
y22-11	TL25-48	Black	rr	TL27-36	Black	r	0	138	

[a] Origin means the maternal colonies where queens and males used for controlled genetic crossing were produced

[c] $\chi^2(1:1)>3.84$ means the ratio is significantly different from 1:1

[b] Genotypes of queen and males were inferred from phenotype segregation among their sisters workers from their maternal colony

Table S3. Specimen information for GWAS.

Sample ID	SEX/ CASTE	T5 color	Worker parent	Origin of worker	DATE COLLECTED	COUNTRY: PROVINCE:CITY	LONGTITUDE	LATITUDE	Altitude/m
R4TL25-47-1	Male	Black	R4TL25-47	TL25-47	2021.5.12	China:GuiZhou: TongRen	107.484283	27.919751	882.6
R4TL25-47-2	Male	Red							
R7TL25-47-6	Male	Red	R7TL25-47						
R7TL25-47-7	Male	Black							
R4TL25-48-3	Male	Black	R4TL25-48	TL25-48	2021.5.12	China:GuiZhou: TongRen	107.484283	27.919751	882.6
R4TL25-48-6	Male	Red							
R6TL25-48-3	Male	Black	R6TL25-48						
R6TL25-48-7	Male	Red							
R4TL27-1-6	Male	Black	R4TL27-1	TL27-1	2021.5.13	China:GuiZhou: ZunYi	106.824411	28.08413	1091.7
R4TL27-1-3	Male	Red							
R6TL27-1-6	Male	Red	R6TL27-1						
R6TL27-1-10	Male	Black							
R1TL27-13-7	Male	Red	R1TL27-13	TL27-13	2021.5.13	China:GuiZhou: ZunYi	106.824411	28.08413	1091.7
R1TL27-13-8	Male	Black							
R2TL27-36-3	Male	Black	R2TL27-36	TL27-36	2021.5.13	China:GuiZhou: ZunYi	106.824411	28.08413	1091.7
R2TL27-36-5	Male	Red							
R4TL27-36-2	Male	Black	R4TL27-36						
R4TL27-36-3	Male	Red							
R7TL27-36-9	Male	Red	R7TL27-36						
R7TL27-36-10	Male	Black							
R1TL27-44-4	Male	Black	R1TL27-44	TL27-44	2021.5.13	China:GuiZhou: ZunYi	106.824411	28.08413	1091.7
R1TL27-44-5	Male	Red							
R8TL27-48-7	Male	Black	R8TL27-48	TL27-48	2021.5.13	China:GuiZhou: ZunYi	106.824411	28.08413	1091.7
R8TL27-48-9	Male	Red							
R17TL27-64-3	Male	Black	R17TL27-64	TL27-64	2021.5.13	China:GuiZhou: ZunYi	106.824411	28.08413	1091.7
R17TL27-64-4	Male	Red							
R14TL27-74-6	Male	Red	R14TL27-74	TL27-74	2021.5.13	China:GuiZhou: ZunYi	106.824411	28.08413	1091.7
R14TL27-74-7	Male	Black							
R16TL27-74-7	Male	Black	R16TL27-74						
R16TL27-74-8	Male	Red							
R4TL25-60-6	Male	Red	R4TL25-60	TL25-60	2021.5.12	China:GuiZhou: TongRen	107.484283	27.919751	882.6
R4TL25-60-5	Male	Black							
R2TL25-14-8	Male	Red	R2TL25-14	TL25-14	2021.5.12	China:GuiZhou: TongRen	107.484283	27.919751	882.6
R1TL25-43-1	Male	Red	R1TL25-43	TL25-43	2021.5.12	China:GuiZhou: TongRen	107.484283	27.919751	882.6
R2TL25-12-2	Male	Red	R2TL25-12	TL25-12	2021.5.12	China:GuiZhou: TongRen	107.484283	27.919751	882.6
R4TL27-14-4	Male	Red	R4TL27-14	TL27-14	2021.5.13	China:GuiZhou: ZunYi	106.824411	28.08413	1091.7
R5TL27-41-7	Male	Red	R5TL27-41	TL27-41	2021.5.13	China:GuiZhou: ZunYi	106.824411	28.08413	1091.7
R5TL27-70-5	Male	Red	R5TL27-70	TL27-70	2021.5.13	China:GuiZhou: ZunYi	106.824411	28.08413	1091.7
B5TL25-47-7	Male	Black	B5TL25-47	TL25-47	2021.5.12	China:GuiZhou: TongRen	107.484283	27.919751	882.6
B2TL25-59-2	Male	Black	B2TL25-59	TL25-59	2021.5.12	China:GuiZhou: TongRen	107.484283	27.919751	882.6
B6TL27-1-4	Male	Black	B6TL27-1	TL27-1	2021.5.13	China:GuiZhou: ZunYi	106.824411	28.08413	1091.7
B4TL27-40-9	Male	Black	B4TL27-40	TL27-40	2021.5.13	China:GuiZhou: ZunYi	106.824411	28.08413	1091.7
B1TL27-44-4	Male	Black	B1TL27-44	TL27-44	2021.5.13	China:GuiZhou: ZunYi	106.824411	28.08413	1091.7
B4TL27-59-1	Male	Black	B4TL27-59	TL27-59	2021.5.13	China:GuiZhou: ZunYi	106.824411	28.08413	1091.7

Table S4. Summary of whole genome sequencing datasets utilized in GWAS.

Sample ID	DNA ID	Phenotype	Total number of clean reads	Average sequencing Depth (Coverage)	Breadth at 1X (%Genome covered)	% reads mapped
R4TL 25-47-1	JXD461	Black	65493638	31.22	98.88%	98.81%
R4TL 25-47-2	JXD462	Orange	74172802	30.66	98.87%	98.90%
R7TL 25-47-6	JXD463	Orange	80356054	38.01	98.93%	98.76%
R7TL 25-47-7	JXD464	Black	102596868	48.2	98.94%	98.87%
R4TL 25-48-3	JXD465	Black	66071836	31.25	98.95%	98.55%
R4TL 26-48-6	JXD466	Orange	60699354	28.78	98.97%	99.19%
R6TL 25-48-3	JXD467	Black	54961082	26.26	98.97%	98.01%
R6TL 25-48-7	JXD468	Orange	66665492	31.88	98.96%	98.83%
R4TL 27-1-6	JXD469	Black	65847916	31.27	98.95%	98.88%
R4TL 27-1-3	JXD470	Orange	70735596	25.01	98.94%	73.57%
R6TL 27-1-6	JXD471	Orange	50893512	24.83	98.94%	98.24%
R6TL 27-1-10	JXD472	Black	57435730	27.65	98.95%	99.11%
R1TL 27-13-7	JXD473	Orange	65091206	29.96	98.97%	98.93%
R1TL 27-13-8	JXD474	Black	61730848	28.95	98.92%	98.89%
R2TL 27-36-3	JXD475	Black	77209858	21.74	98.93%	58.66%
R2TL 27-36-5	JXD476	Orange	54088432	25.36	98.96%	99.15%
R4TL 27-36-2	JXD477	Black	52483540	24.93	98.90%	98.76%
R4TL 27-36-3	JXD478	Orange	53459584	25.69	98.93%	99.07%
R7TL 27-36-9	JXD479	Orange	85062748	36.39	98.95%	90.62%
R7TL 27-36-10	JXD480	Black	53395704	24.9	98.92%	98.01%
R1TL 27-44-4	JXD481	Black	71370516	32.8	98.94%	98.99%
R1TL 27-44-5	JXD482	Orange	57092434	26.92	98.95%	97.52%
R8TL 27-48-7	JXD483	Black	64263194	30.72	98.96%	99.28%
R8TL 27-48-9	JXD484	Orange	65203132	30.18	98.96%	99.05%
R17TL 27-64-3	JXD485	Black	101842470	47.94	98.95%	98.96%
R17TL 27-64-4	JXD486	Orange	65338570	31.44	98.91%	98.96%
R14TL 27-74-6	JXD487	Orange	58883168	21.58	98.90%	77.26%
R14TL 27-74-7	JXD488	Black	57355480	26.97	98.91%	98.85%
R16TL 27-74-7	JXD489	Black	50879760	24.3	98.90%	99.04%
R16TL 27-74-8	JXD490	Orange	45574790	21.72	98.93%	99.11%
R4TL 25-60-6	JXD491	Orange	48335054	22.58	98.95%	98.77%
R4TL 25-60-5	JXD492	Black	42293008	19.76	98.92%	95.06%
R2TL 25-14-8	JXD493	Orange	27628160	13.87	98.78%	98.53%
R1TL 25-43-1	JXD494	Orange	57532542	26.23	98.91%	97.47%
R2TL 25-12-2	JXD495	Orange	52445296	24.65	98.95%	98.72%
R2TL 27-14-4	JXD496	Orange	54938434	25.97	98.95%	98.93%
R5TL 27-41-7	JXD497	Orange	44447066	21.21	98.89%	98.13%
R5TL 27-70-5	JXD498	Orange	48836446	23.45	98.94%	98.92%
B5TL 25-47-7	JXD499	Black	56679432	26.85	98.89%	98.85%
B2TL 25-59-2	JXD500	Black	21461798	11	98.71%	99.22%
B6TL 27-1-4	JXD501	Black	56457130	26.52	98.93%	98.62%
B4TL 27-40-9	JXD502	Black	55911204	23.18	98.91%	98.49%
B1TL 27-44-4	JXD503	Black	58311900	27.23	98.94%	99.09%
B4TL 27-59-1	JXD504	Black	58710962	23.91	98.91%	85.46%

Table S5. Specimen information for genotyping.

SAMPLE ID	DNA ID	SPECIES	T5 COLOR	LATITUDE	LONGTITUDE	DATE COLLECTED	COUNTRY:PROVINCE	SEX/CASTE
TL21 6-1w	JXD56	*B.breviceps*	Black	29.788851	120.935009	2021.4.20	China:ZheJiang	worker
TL21 7-1w	JXD57	*B.breviceps*	Black	28.942712	120.550302	2021.4.21	China:ZheJiang	worker
CYX21 8-1w	JXD58	*B.breviceps*	Black	28.919721	120.558682	2021.5.3	China:ZheJiang	worker
CYX21 11-2w	JXD59	*B.breviceps*	Black	26.667645	119.199879	2021.5.6	China:FuJian	worker
CYX21 115-1	JXD60	*B.breviceps*	Orange	29.418934	95.406946	2021.8.10	China:XiZang	worker
CYX21 116-1	JXD61	*B.breviceps*	Orange	29.406729	95.383694	2021.8.10	China:XiZang	worker
TL21 25-19	JXD62	*B.breviceps*	Black	27.919751	107.484283	2021.5.12	China:GuiZhou	worker
TL21 25-21	JXD63	*B.breviceps*	Black	27.919751	107.484283	2021.5.12	China:GuiZhou	worker
TL21 26-1	JXD64	*B.breviceps*	Orange	27.770578	107.182518	2021.5.12	China:GuiZhou	worker
TL21 27-4	JXD65	*B.breviceps*	Orange	28.08413	106.824411	2021.5.13	China:GuiZhou	worker
TL21 27-?	JXD66	*B.breviceps*	Black	28.08413	106.824411	2021.5.13	China:GuiZhou	worker
TL21 54-8	JXD72	*B.breviceps*	Black	26.409002	107.776611	2021.7.25	China:GuiZhou	worker
TL21 55-10	JXD73	*B.breviceps*	Orange	26.406905	106.886001	2021.7.26	China:GuiZhou	worker
TL21 56-3	JXD74	*B.breviceps*	Black	26.346969	106.897178	2021.7.26	China:GuiZhou	worker
TL21 57-1	JXD76	*B.breviceps*	Black	26.32583	106.904734	2021.7.26	China:GuiZhou	worker
TL21 58-1	JXD78	*B.breviceps*	Black	26.301715	106.941361	2021.7.26	China:GuiZhou	worker
TL21 61-5	JXD80	*B.breviceps*	Orange	26.243166	107.08122	2021.7.26	China:GuiZhou	worker
TL21 67-1	JXD82	*B.breviceps*	Orange	25.459789	106.164884	2021.7.26	China:GuiZhou	worker
TL21 26-7	JXD85	*B.breviceps*	Black	27.770578	107.182518	2021.5.12	China:GuiZhou	worker
TL21 25-27	JXD87	*B.breviceps*	Black	27.919751	107.484283	2021.5.12	China:GuiZhou	worker
TL21 25-32	JXD88	*B.breviceps*	Orange	27.919751	107.484283	2021.5.12	China:GuiZhou	worker
NA	JXD89	*B.breviceps*	Orange	29.83927	106.391342	2017.8.20	China:ChongQin	worker
NA	JXD91	*B.breviceps*	Orange	28.27272	105.631271	2013.8.30	China:SiChuan	worker
NA	JXD93	*B.breviceps*	Black	28.27272	105.631271	2013.8.30	China:SiChuan	worker
NA	JXD95	*B.breviceps*	Orange	29.93305	103.03527	2019.6.22	China:SiChuan	worker
NA	JXD97	*B.breviceps*	Orange	31.060049	103.719101	*NA*	China:SiChuan	worker
NA	JXD98	*B.breviceps*	Orange	29.58944	103.385	2019.6.26	China:SiChuan	worker
CYX21 4-33	JXD99	*B.breviceps*	Orange	31.0268	103.5782	2021.3.17	China:SiChuan	worker
CYX21 4-13	JXD100	*B.breviceps*	Orange	31.0268	103.5782	2021.3.17	China:SiChuan	worker
TL20-167	JXD101	*B.breviceps*	Black	29.646525	120.007412	2020.10.1	China:ZheJiang	worker
TL20-144	JXD102	*B.breviceps*	Black	27.647273	117.919493	2020.9.26	China:FuJian	worker
NA	JXD103	*B.breviceps*	Black	24.723221	114.2564	2020.9.13	China:GuangDong	worker
NA	JXD104	*B.breviceps*	Black	29.074	117.5841	2020.6.7	China:JiangXi	worker
CYX21 33-5	JXD105	*B.breviceps*	Orange	24.634072	102.919918	2021.4.13	China:YunNan	worker
CYX21 35-14	JXD106	*B.breviceps*	Orange	24.894577	103.040373	44304	China:YunNan	worker
TL20-28	JXD107	*B.breviceps*	Orange	25.42213	98.662387	2020.8.29	China:YunNan	worker
TL20-47	JXD108	*B.breviceps*	Orange	23.901264	100.091313	2020.8.31	China:YunNan	worker
TL20-54	JXD109	*B.breviceps*	Orange	24.696221	102.750645	2020.9.4	China:YunNan	worker
WYF20 2-1	JXD110	*B.breviceps*	Orange	25.639433	100.210144	2020.8.6	China:YunNan	worker
TL21 73-1	JXD111	*B.breviceps*	Black	25.814426	107.309741	2021.7.27	China:GuiZhou	worker
CYX21 94-1	JXD113	*B.breviceps*	Black	26.642262	106.463416	2021.7.30	China:GuiZhou	worker
CYX21 98-3	JXD116	*B.breviceps*	Orange	26.315664	105.638881	2021.7.31	China:GuiZhou	worker
CYX21 100-1	JXD117	*B.breviceps*	Black	26.237729	105.395136	2021.7.31	China:GuiZhou	worker
CYX21 101-1	JXD119	*B.breviceps*	Black	26.64879	104.932453	2021.8.1	China:GuiZhou	worker
CYX20-806	JXD120	*B.breviceps*	Orange	29.078889	107.055	2020.8.15	China:ChongQin	worker
TL21 32-6	JXD123	*B.breviceps*	Orange	28.714493	108.792659	2021.5.19	China:ChongQin	worker
CYX21 40-2	JXD124	*B.breviceps*	Orange	32.504162	105.625231	2021.6.21	China:SiChuan	worker
CYX21 45-1	JXD125	*B.breviceps*	Orange	32.657169	105.921777	2021.6.24	China:SiChuan	worker
CYX21 47-1	JXD126	*B.breviceps*	Orange	32.843092	106.327221	2021.6.24	China:ShanXi	worker
CYX21 61-1	JXD128	*B.breviceps*	Orange	33.230136	106.973494	2021.6.26	China:ShanXi	worker
CYX21 62-1	JXD129	*B.breviceps*	Orange	33.1916	107.04	2021.6.27	China:ShanXi	worker
CYX21 64-1	JXD130	*B.breviceps*	Orange	33.186954	107.030284	2021.6.27	China:ShanXi	worker
CYX21 41-1	JXD131	*B.breviceps*	Orange	33.04737	105.256236	2021.6.21	China:GanSu	worker
CYX21 43-1	JXD132	*B.breviceps*	Orange	32.755458	105.323954	2021.6.23	China:GanSu	worker
CYX21 44-1	JXD133	*B.breviceps*	Orange	32.773411	105.413661	2021.6.23	China:GanSu	worker
TL20-243	JXD134	*B.breviceps*	Black	29.66152	120.008814	2020.10.1	China:ZheJiang	worker
TL20-248	JXD135	*B.breviceps*	Black	30.321209	119.512109	2020.10.2	China:ZheJiang	worker
TL20-263	JXD136	*B.breviceps*	Black	28.028308	120.236592	2020.9.29	China:ZheJiang	worker

SAMPLE ID	DNA ID	*SPECIES*	T5 COLOR	*LATITUDE*	*LONGTITUDE*	*DATE COLLECTED*	COUNTRY:PROVINCE	SEX/CASTE
TL20-231	JXD137	*B.breviceps*	Black	28.909647	120.441704	2020.9.30	China:ZheJiang	worker
TL20-235	JXD138	*B.breviceps*	Black	29.340131	120.768735	2020.9.30	China:ZheJiang	worker
TL21 3-1	JXD139	*B.breviceps*	Black	29.792879	120.928674	2021.4.19	China:ZheJiang	worker
CYX21 85-1	JXD141	*B.breviceps*	Black	27.672761	118.0148	2021.7.13	China:FuJian	worker
CYX21 92-1	JXD142	*B.breviceps*	Black	25.855287	116.307279	2021.7.21	China:FuJian	worker
CYX21 88-2	JXD143	*B.breviceps*	Black	24.767675	113.59321	2021.7.18	China:GuangDong	worker
TL21 36-1	JXD144	*B.breviceps*	Black	26.038967	110.148947	2021.7.22	China:GuangXi	worker
TL21 77-1	JXD145	*B.breviceps*	Black	24.742955	110.478489	2021.7.30	China:GuangXi	worker
NA	JXD146	*B.breviceps*	Black	24.360601	111.230141	2020.8.25	China:GuangXi	worker
TL21 39-4	JXD147	*B.breviceps*	Black	26.151334	110.16438	2021.7.22	China:HuNan	worker
TL21 41-1	JXD148	*B.breviceps*	Orange	26.166167	110.174566	2021.7.23	China:HuNan	worker
CYX21 67-1	JXD149	*B.breviceps*	Orange	30.659956	111.147727	2021.7.4	China:HuBei	worker
CYX21 68-1	JXD150	*B.breviceps*	Orange	30.860807	110.916691	2021.7.4	China:HuBei	worker
CYX21 69-1	JXD151	*B.breviceps*	Orange	30.871187	110.912561	2021.7.4	China:HuBei	worker
CYX21 72-1	JXD152	*B.breviceps*	Orange	31.171914	110.275868	2021.7.5	China:HuBei	worker
CYX21 73-4	JXD153	*B.breviceps*	Orange	31.365729	110.241895	2021.7.5	China:HuBei	worker
CYX21 77-1	JXD154	*B.breviceps*	Orange	31.365006	110.242596	2021.7.6	China:HuBei	worker
CYX21 82-1	JXD155	*B.breviceps*	Black	28.034974	117.33934	2021.7.12	China:JiangXi	worker
CYX21 83-1	JXD156	*B.breviceps*	Black	28.284734	117.423351	2021.7.13	China:JiangXi	worker
CYX21 84-5	JXD157	*B.breviceps*	Black	28.358536	117.456439	2021.7.13	China:JiangXi	worker
CYX21 90-1	JXD158	*B.breviceps*	Black	25.908904	114.894075	2021.7.21	China:JiangXi	worker
CYX21 33-1	JXD159	*B.breviceps*	Orange	24.634072	102.919918	2021.4.13	China:YunNan	worker
CYX21 35-10	JXD160	*B.breviceps*	Orange	24.894577	103.040373	2021.4.18	China:YunNan	worker
TL20-316	JXD161	*B.breviceps*	Orange	24.818468	99.602011	2020.8.30	China:YunNan	worker
TL20-320	JXD162	*B.breviceps*	Orange	23.871011	100.074843	2020.9.1	China:YunNan	worker
TL20-321	JXD163	*B.breviceps*	Orange	24.63193	102.754434	2020.9.4	China:YunNan	worker
TL20-322	JXD164	*B.breviceps*	Orange	24.922515	103.04885	2020.9.4	China:YunNan	worker
TL20-324	JXD165	*B.breviceps*	Orange	25.043161	102.600834	2020.9.5	China:YunNan	worker
TL20-326	JXD166	*B.breviceps*	Orange	25.650291	100.28351	2020.9.7	China:YunNan	worker
TL20-328	JXD167	*B.breviceps*	Orange	25.65529	100.288906	2020.9.7	China:YunNan	worker
ZXJ21 1-1	JXD169	*B.breviceps*	Black	25.639433	100.210144	2021.8.14	China:YunNan	worker
ZXJ21 2-1	JXD170	*B.breviceps*	Orange	25.911882	103.282593	2021.8.14	China:YunNan	worker
TL20-311	JXD171	*B.breviceps*	Orange	25.216956	98.495276	2020.8.28	China:YunNan	worker
b411	JXD172	*B.breviceps*	Orange	*NA*	*NA*	*NA*	Thailand:*NA*	worker
b410	JXD173	*B.breviceps*	Orange	*NA*	*NA*	*NA*	Burma:*NA*	worker
CYX20-856	C65	*B.breviceps*	Orange	31.018889	103.59	2020.8.15	China:SiChuan	worker
CYX20-857	C66	*B.breviceps*	Orange	31.045	103.52833	2020.8.15	China:SiChuan	worker
CYX20-858	C67	*B.breviceps*	Orange	30.5675	103.25778	2020.8.16	China:SiChuan	worker
CYX20-859	C68	*B.breviceps*	Orange	30.63583	103.40805	2020.8.17	China:SiChuan	worker
CYX20-860	C69	*B.breviceps*	Orange	30.076388	103.0561	2020.8.18	China:SiChuan	worker
CYX20-861	C70	*B.breviceps*	Orange	29.57321	103.461476	2020.8.19	China:SiChuan	worker
CYX20-862	C71	*B.breviceps*	Orange	29.52666	103.4322	2020.8.19	China:SiChuan	worker
CYX20-863	C72	*B.breviceps*	Orange	29.543055	103.41166	2020.8.17	China:SiChuan	worker
CYX20-865	C74	*B.breviceps*	Black	25.644015	109.915342	2020.8.25	China:GuangXi	worker
CYX20-866	C75	*B.breviceps*	Black	25.869917	110.47362	2020.8.25	China:GuangXi	worker
CYX20-867	C76	*B.breviceps*	Black	27.71352	117.963926	2020.9.26	China:FuJian	worker
CYX20-868	C77	*B.breviceps*	Black	27.71352	117.963926	2020.9.26	China:FuJian	worker
CYX20-869	C78	*B.breviceps*	Black	27.633954	117.919599	2020.9.27	China:FuJian	worker
CYX20-870	C79	*B.breviceps*	Black	26.681216	119.201944	2020.9.28	China:FuJian	worker
CYX20-871	C80	*B.breviceps*	Black	27.695966	120.287937	2020.9.29	China:ZheJiang	worker
CYX20-872	C81	*B.breviceps*	Black	27.825158	120.292712	2020.9.29	China:ZheJiang	worker
CYX20-873	C82	*B.breviceps*	Orange	29.340131	120.768735	2020.9.30	China:ZheJiang	worker
CYX20-874	C83	*B.breviceps*	Black	29.646525	120.007412	2020.10.1	China:ZheJiang	worker
CYX20-875	C84	*B.breviceps*	Black	30.203021	119.589566	2020.10.1	China:ZheJiang	worker
CYX20-877	C86	*B.breviceps*	Orange	25.42213	98.662387	2020.8.29	China:YunNan	worker
CYX20-878	C87	*B.breviceps*	Orange	25.224215	98.594676	2020.8.29	China:YunNan	worker
CYX20-879	C88	*B.breviceps*	Orange	25.102194	98.549019	2020.8.29	China:YunNan	worker
CYX20-880	C89	*B.breviceps*	Orange	24.818468	99.602011	2020.8.30	China:YunNan	worker
CYX20-881	C90	*B.breviceps*	Orange	23.871011	100.074843	2020.9.1	China:YunNan	worker
CYX20-882	C91	*B.breviceps*	Orange	23.85827	100.08104	2020.9.1	China:YunNan	worker
CYX20-883	C92	*B.breviceps*	Orange	24.63193	102.754434	2020.9.4	China:YunNan	worker

SAMPLE ID	DNA ID	*SPECIES*	T5 COLOR	*LATITUDE*	*LONGTITUDE*	*DATE COLLECTED*	COUNTRY:PROVINCE	SEX/CASTE
CYX20-884	C93	*B.breviceps*	Orange	24.968428	103.019013	2020.9.4	China:YunNan	worker
CYX20-885	C94	*B.breviceps*	Orange	24.959967	102.680245	2020.9.5	China:YunNan	worker
CYX20-886	C95	*B.breviceps*	Orange	25.68722	100.433969	2020.9.7	China:YunNan	worker
CYX20-887	C96	*B.breviceps*	Orange	25.683281	100.302834	2020.8.15	China:YunNan	worker
CYX20-888	C97	*B.breviceps*	Orange	25.683281	100.302834	2020.8.15	China:YunNan	worker
NA	JXD187	*B.kashmirensis*	Orange	*NA*	*NA*	*NA*	China:QingHai	worker
NA	JXD188	*B.kashmirensis*	Orange	*NA*	*NA*	*NA*	China:QingHai	worker
cyx22-14-4	yd83	*B. haemorrhoidalis*	Black	*NA*	*NA*	*NA*	China:YunNan	worker
cyx22-14-6	yd85	*B. haemorrhoidalis*	Black	*NA*	*NA*	*NA*	China:YunNan	worker
cyx22-14-8	yd86	*B. haemorrhoidalis*	Black	*NA*	*NA*	*NA*	China:YunNan	worker
cyx22-19-1	yd93	*B. haemorrhoidalis*	Black	*NA*	*NA*	*NA*	China:YunNan	worker
cyx22-19-5	yd94	*B. haemorrhoidalis*	Black	*NA*	*NA*	*NA*	China:YunNan	worker
cyx22-19-6	yd95	*B. haemorrhoidalis*	Black	*NA*	*NA*	*NA*	China:YunNan	worker
NA	*NA*	*B.grahami*	Orange	*NA*	*NA*	*NA*	*NA*	worker
NA	*NA*	*B.grahami*	Orange	*NA*	*NA*	*NA*	*NA*	worker
NA	JXD234r	*B.montivagus*	Orange	*NA*	*NA*	*NA*	NA	worker
NA	JXD182r	*B.montivagus*	Orange	*NA*	*NA*	*NA*	NA	worker
NA	JXD183r	*B.montivagus*	Orange	*NA*	*NA*	*NA*	NA	worker
NA	JXD184r	*B.montivagus*	Orange	*NA*	*NA*	*NA*	NA	worker
NA	JXD254b	*B.montivagus*	Black	*NA*	*NA*	*NA*	NA	worker
NA	JXD311b	*B.montivagus*	Black	*NA*	*NA*	*NA*	NA	worker
NA	JXD252b	*B.montivagus*	Black	*NA*	*NA*	*NA*	NA	worker

Table S6. Primers used for genotyping to narrow down the locus

Primer Name	Forward (5'-3')	Reverse (5'-3')	~Size (bp)
F1R1	ATGGAAGGAAGATACTGGGAATCG	GGTACGATGATGTTCCGAGTTAGA	1,048
F2R2	GAATAGCCTGGAGAAAGATCGCTA	CATAACGGTGTAGCTCTCGTTTTC	1,380
F3R3	AGGGTGAACGGTGGTAATATCTTC	ACGATAGGTCAGAGAGAAAATCGG	769
F4R4	GTTATCCCGCTGGAGAAATTCTTG	GATTGCTGAGAGTAATCCTTTGCC	1,228
F7R7	GATCCAAGAAGCAATTCCTATGCC	CGAGTGTTTCATCGCTATGTAACC	1,502
F9R9	GCTTGTTACATAGTTCCACGCTAC	CTCTGTCGTTAACGCGAGATAGTA	812
F12R12	GTTAATTGTCTCCGCGTTACACAC	CGCTCTCTAGTTTCGTTCAACAAC	798
F13R13	CTCAATATCCGCGTGTAAAGTCTC	CTGGAATCGATGCAATGAGTTCC	766
F14R14	GATCGCTCATCGTATCGATTTCCT	TTAATTGAGAAGTCCTAGACGCCC	779
F25R25	CAAGAATTTCTCCAGCGGGATAAC	GGATCTGGTTTTGCTACCTTGATC	1,528
F29R29	CGTTTCATGTTCGAATTCCAGTGG	CCCGATTAAACGTAACTCGGTAGA	1,486
F30R30	ATTAAGCATCGGTGTAGTTGAGCC	GTCACGAAGCTGTATGCATTCTAC	1,455
F31R31	GGCATAGGAATTGCTTCTTGGATC	CTCGAAGGATTAATCGAAGGATCG	1,450
F32R32	GAAGCACTCGCGAATTCGATATTC	CCGAAGTACGAGTAATATCCGTGT	1,363
F33R33	CTCGATCGTTGCATTTAGAAGACG	GAACGAATTCCTGTCGCTGTTTAC	1,344
F34R34	AGACGAGAGATCATTAGCGCATAG	GGCAAAGGATTACTCTCAGCAATC	1,283
F35R35	GAGTTCCTCTCTGTAGACAACCTC	GTTACATAGCGATGAAACACTCGG	1,194

Table S7. Primers used for genotyping the 1.4-kb color locus

Species	Primer Name	Forward (5'-3')	Reverse (5'-3')	~Size (bp)
B. breviceps	F42R42	CTTTTTCTTCCCAGTCTTCCAACC	GGTAGAACGACCGTGTGTACAATA	884
B. grahami	F43R43	CCACGTAGTCAGGTACAATTACGA	CGAATTTCTTCGCTTGTTGC	980
B. montivagus	F42R42	CTTTTTCTTCCCAGTCTTCCAACC	GGTAGAACGACCGTGTGTACAATA	884
	F44R44	AAAGACGATCACGCGCGGAG	AATCCTCGGAGTAAGACTGGTT	1144
B.haemorrhoidalis	F107R107	TTGCGTACCCTCGTTCTCTC	TCCGCGTACGATTGTATTATAAGT	846bp
	F108R108	ACAATCGTACGCGGATTCTAA	TTCCCGGTGCTAGCAAATCT	811bp

Table. S8. Specimen used for transcriptome sequencing

SAMPLE ID	INDIVIDUAL ID	Colony	T5 COLOR	LATITUDE	LONGTITUDE	COUNTRY: PROVINCE	SEX/ CASTE
R9Q	r9Q-1	TL25-14	Orange	107.48428	27.919751	China:GuiZhou	worker
	r9Q-2	TL25-14	Orange	107.48428	27.919751	China:GuiZhou	worker
	r9Q-3	TL25-14	Orange	107.48428	27.919751	China:GuiZhou	worker
R10Q	r10Q-1	TL27-71	Orange	106.82441	28.08413	China:GuiZhou	worker
	r10Q-2	TL27-71	Orange	106.82441	28.08413	China:GuiZhou	worker
	r10Q-3	TL27-71	Orange	106.82441	28.08413	China:GuiZhou	worker
R11Q	r11Q-1	TL27-75	Orange	106.82441	28.08413	China:GuiZhou	worker
	r11Q-2	TL27-75	Orange	106.82441	28.08413	China:GuiZhou	worker
	r11Q-3	TL27-75	Orange	106.82441	28.08413	China:GuiZhou	worker
B6Q	b6Q-1	TL25-25	Black	107.48428	27.919751	China:GuiZhou	worker
	b6Q-2	TL25-25	Black	107.48428	27.919751	China:GuiZhou	worker
	b6Q-3	TL25-25	Black	107.48428	27.919751	China:GuiZhou	worker
B7Q	b7Q-1	TL27-40	Black	106.82441	28.08413	China:GuiZhou	worker
	b7Q-2	TL27-40	Black	106.82441	28.08413	China:GuiZhou	worker
	b7Q-3	TL27-40	Black	106.82441	28.08413	China:GuiZhou	worker
B8Q	b8Q-1	TL27-59	Black	106.82441	28.08413	China:GuiZhou	worker
	b8Q-2	TL27-59	Black	106.82441	28.08413	China:GuiZhou	worker
	b8Q-3	TL27-59	Black	106.82441	28.08413	China:GuiZhou	worker

Table. S9. Summary of transcriptomic sequencing data

Sample ID	Raw Reads	Raw Bases(G)	Clean Reads	Clean Bases (G)	Reads with UIDs	Dedup Reads	total mapped(%)
B6_Q_rep1	61779546	9.27	52344768	7.63	50790898(97.03%)	41519378(79.32%)	40013091(96.37)
B7_Q_rep2	38659802	5.8	29733314	4.41	28793444(96.84%)	23627008(79.46%)	23048174(97.55)
B8_Q_rep3	71102522	10.67	60857336	8.77	59056124(97.04%)	47999904(78.87%)	45933217(95.69)
R10_Q_rep2	38164136	5.72	32359414	4.65	27859458(86.09%)	22495934(69.52%)	21775570(96.80)
R11_Q_rep3	60494816	9.07	52628974	7.61	50858748(96.64%)	41239060(78.36%)	39658138(96.17)
R9_Q_rep1	42834694	6.43	37742166	5.48	36395076(96.43%)	28870770(76.49%)	27827186(96.39)

Table. S10. Primers used for validating ncRNA reads detected from the transcriptome

Primer Name	Forward (5'-3')	Reverse (5'-3')	~Size (bp)
F102.1R102	CTTGAGATACAGTCCAAAACAGCC	GAAGAAGAGTGGAATGGCAAGAAG	160
F102.2R102	CCTTGAGATACAGTCCAAAACAGC	GAAGAAGAGTGGAATGGCAAGAAG	161
F102.3R102	GCCTTGAGATACAGTCCAAAACAG	GAAGAAGAGTGGAATGGCAAGAAG	162
F102.3R102.1	GCCTTGAGATACAGTCCAAAACAG	CCCCTTTGCTGTTATAGTTGCTG	260
F102.3R102.2	GCCTTGAGATACAGTCCAAAACAG	CGCATACCCCTTTGCTGTTATAGT	261
F102.3R102.3	GCCTTGAGATACAGTCCAAAACAG	TCGCATACCCCTTTGCTGTTATAG	262

Table. S11. Primers used for quantitative real-time PCR

Primer Name	Forward (5'-3')	Reverse (5'-3')	Target gene
F105.1/R105.1	GTCCAAAACAGCCAAACAATGC	GAAGAAATTCCAAAGGAACCCGTG	lncRNA-iab6
F-abdA2/R-abdA2	GATGAAGCTGAAGAAGGAGCTGAG	CTTCTTCATCATGTCCTGCTCCTC	*abd-A*
F-abdB/R-abdB	GAACCTGTTACCTCCAGTCCGTA	TTTCTTTCGTACCGTCACCTGAC	*Abd-B*
F-BbvATPase/R-BbvATPase	GGCAATCATATCAACAGTCTCA	TGCATCCCGAACACTAGCATC	*v-ATPase*

Supplementary References

Ge, J., Zhou, X., Ge, Z., et al. 2021. Phased contests allow rapid hierarchy formation in paired bumble bee workers. *Anim Behav.* 179: 125-138.

Hines, H. M., Counterman, B. A., Papa R., et al. 2011. Wing patterning gene redefines the mimetic history of *Heliconius* butterflies. *Proc Natl Acad Sci U.S.A.* 108(49): 19666-19671.

Owen, R. E. & Plowright, R. C. 1980. Abdominal pile color dimorphism in the bumble bee, *Bombus melanopygus*. *J Hered.* 71: 241-247.

Princen, S. A., Van Oystaeyen, A., Van Zweden, et al. 2020. Worker dominance and reproduction in the bumble bee *Bombus terrestris:* when does it pay to bare one's mandibles? *Anim Behav.* 166: 41-50.

Williams, P. 2007. The distribution of bumblebee colour patterns worldwide: Possible significance for thermoregulation, crypsis, and warning mimicry. *Biol J Linn Soc.* 92(1): 97-118.

Williams, P. H. 2012. The distribution of bumblebee colour patterns worldwide: Possible significance for thermoregulation, crypsis, and warning mimicry. *Biol J Linn Soc Lond.* 92(1): 97-118.

Williams, P. H. 2022. The bumble bees of the Himalaya–An Identification Guide. Abc Taxa.
